# Platelet Functional Profile Is Altered in Metabolic Dysfunction‐Associated Steatotic Liver Disease

**DOI:** 10.1111/liv.70231

**Published:** 2025-07-28

**Authors:** Marco Castelli, Mirko Zoncapè, Alessandra Meneguzzi, Anna Mantovani, David Sacerdoti, Pietro Minuz, Andrea Dalbeni

**Affiliations:** ^1^ Department of Medicine, Medicine C University of Verona and Hospital Trust of Verona (Azienda Ospedaliera Universtaria AOUI) Verona Italy; ^2^ Liver Unit, Department Medicine University of Verona Verona Italy

**Keywords:** cirrhosis, fibrogenesis, haemostasis, liver fibrosis, metabolic dysfunction‐associated steatotic liver disease, platelet activation, soluble P‐selectin, thromboxane B2

## Abstract

**Background and Aims:**

Metabolic dysfunction‐associated steatotic liver disease (MASLD) is a leading cause of chronic liver disease, often progressing to fibrosis and up to cirrhosis. Platelet activation plays a potential role in MASLD progression, but its functional profile across disease stages remains unclear. This study aimed to characterise the platelet functional profile and phenotype in patients with MASLD ≤ F2 and F3–F4 fibrosis compared to healthy controls.

**Methods:**

We analysed biomarkers of in vivo platelet activation, including urinary 11‐dehydro‐thromboxane B2 (11‐dh‐TXB_2_) and plasma soluble P‐selectin. Platelet phenotype was assessed via microfluidic assays and flow cytometry, evaluating adhesion and fibrinogen receptor activation and Annexin V. Patients were stratified according to liver stiffness measurement (LSM) into low fibrosis MASLD (≤ F2) and advanced fibrosis/cirrhosis (F3–F4).

**Results:**

Urinary 11‐dh‐TXB_2_ was comparable between control and the ≤ F2 patients (median difference: 7365 ng/g creatinine, 95% CI: 5257–8764); higher values were observed in F3–F4 patients, with levels significantly elevated compared to controls (6931 ng/g creatinine, 95% CI: 5290–8363). Plasma soluble P‐selectin followed a similar trend. Platelet phenotype MASLD ≤ F2 patients exhibited increased adhesiveness (median difference: 289.8 a.u., 95% CI: 195.8–458.2) and displayed procoagulant activity through binding of Annexin V, whereas F3–F4 patients showed increased adhesiveness (median difference: 361.1 a.u., 95% CI: 163.4–530.4) and reduced fibrinogen receptor activation.

**Conclusions:**

Platelet activation markers correlate with fibrosis severity, highlighting their potential role in disease progression. MASLD ≤ F2 is associated with increased platelet adhesiveness, whereas F3–F4 is characterised by impaired platelet reactivity despite high activation markers. These findings support the involvement of platelets in MASLD fibrosis progression and suggest their potential as biomarkers or therapeutic targets.

**Impact and Implications:**

This study provides a robust scientific rationale for further exploring platelet dynamics as both biomarkers and potential therapeutic targets in MASLD, by demonstrating distinct platelet functional profiles between early (MASLD ≤ F2) and advanced fibrosis (F3–F4). The observed increase in platelet adhesiveness and procoagulant activity in early MASLD, contrasted with a reduced reactivity in advanced stages despite elevated activation markers, is critical for clinicians and researchers aiming to refine risk stratification and optimise treatment strategies. These findings could be practically applied by guiding physicians in monitoring platelet activation markers to identify patients at higher risk of fibrosis progression and by informing the development of targeted antiplatelet interventions. However, given the cross‐sectional design of the study and its inherent methodological limitations, these implications warrant cautious interpretation and further validation through longitudinal research.

**Trial Registration:**

1612CESC

**Summary**

- Urinary 11‐dh‐TXB_2_ and plasma sP‐selectin are particularly increased in F3–F4: these biomarkers indicate in vivo platelet activation and correlate with liver fibrosis severity.
- Both MASLD ≤ F2 and F3–F4 are associated with increased platelet adhesiveness.
- Distinct platelet functional profiles emerge across MASLD ≤ F2 shows a rise in procoagulant platelets, whereas F3–F4 is characterised by reduced fibrinogen receptor activation.
- Platelets may contribute to MASLD fibrosis progression: their role in fibrogenesis and hepatocellular carcinoma development suggests potential as biomarkers or therapeutic targets.

**Abbreviations**
11‐dh‐TXB_2_
: urinary 11‐dehydrothromboxane B2
ACS: acute coronary syndrome
AGILE 3+/4: algorithm based liver fibrosis scores
ALP: alkaline phosphatase
ALT: alanine aminotransferase
APC: allophycocyanin
APRI: AST to platelet ratio index
AST: aspartate aminotransferase
BMI: body mass index
BSA: bovine serum albumin
CAP: controlled attenuation parameter
CD14: monocyte/macrophage marker
CD41a: integrin‐b platelet marker
CD62p: platelet activation marker (P‐selectin)
CD66: neutrophil marker
CI: confidence interval
CKD‐EPI: chronic kidney disease epidemiology collaboration equation
CTR: control group
CVD: cardiovascular disease
DM: type 2 diabetes mellitus
ELISA: enzyme‐linked immunosorbent assay
FACS: fluorescence‐activated cell sorting
FAST: fibrosis‐4 index‐based algorithm
FIB‐4: fibrosis‐4 index
FITC: fluorescein isothiocyanate
GGT: gamma‐glutamyl transferase
Hb: haemoglobin
HCT: haematocrit
HDL: high‐density lipoprotein
HSCs: hepatic stellate cells
IFGT: impaired fasting glucose tolerance
IPF: immature platelet fraction in the platelet count
IRF: immature reticulate fraction in the reticulocytes count
ITAM: immunoreceptor tyrosine‐based activation motif
LDL: low‐density lipoprotein
LSM: liver stiffness measurement
LYMPH: lymphocytes
MALSD ≤ F2: MASLD with low liver fibrosis
MASH: metabolic dysfunction‐associated steatohepatitis
MASLD: metabolic dysfunction‐associated steatotic liver disease
MASLD F3–F4: MASLD with advanced liver fibrosis/cirrhosis
MCV: mean corpuscular volume
MONO: monocytes
MPV: mean platelet volume
NAFLD: non‐alcoholic fatty liver disease
NASH: non‐alcoholic steatohepatitis
NEU: neutrophils
NFS: NAFLD fibrosis score
PAC‐1: platelet activation complex 1
PDGF: platelet‐derived growth factor
PDW: platelet distribution width
PE: phycoerythrin
PLT: platelet count
PPP: platelet‐poor plasma
PRP: platelet‐rich plasma
PS: phosphatidylserine
RBC: red blood cell count
RET: reticulocytes
sP‐selectin: soluble P‐selectin
TGF‐β: transforming growth factor‐beta
TRAP‐6: thrombin receptor‐activating peptide‐6
TXA_2_
: thromboxane A2
VCTE: vibration‐controlled transient elastography
WBC: white blood cell count

## 1 Introduction

Metabolic dysfunction‐associated steatotic liver disease (MASLD), formerly known as non‐alcoholic fatty liver disease (NAFLD), is nowadays the most common chronic liver condition, affecting both adults and children [1, 2, 3].

The prevalence of MASLD has shown a marked increase over recent decades, rising from approximately 18% in 1990 to over 30% today, and it is still progressively increasing. MASLD is particularly common among overweight and obese individuals, affecting around 50% of this group and is even more prevalent (reaching nearly 60%) in those with type 2 diabetes mellitus (DM) [4, 5].

MASLD represents a spectrum of different liver conditions, ranging from simple steatosis to various grades of inflammation (defining the metabolic dysfunction‐associated steatohepatitis [MASH]), and eventually leading to the development of hepatic fibrosis. This may progress to advanced liver fibrosis and cirrhosis (classified as F3 and F4, respectively, using the METAVIR scoring system), which represent the final stage of liver disease, characterised by complete fibrotic disruption of the liver [3, 6].

The pathogenesis of MASLD is closely linked to metabolic and cardiovascular diseases, including hypertension, dyslipidaemia, diabetes mellitus, obesity and metabolic syndrome [7]. The progression from MASLD to MASH and advanced liver fibrosis/cirrhosis (F3–F4) involves a cascade of inflammatory and fibrogenic events, where platelet activation may have a central role, mediating pro‐inflammatory and pro‐fibrotic responses and the haemostatic balance [7].

In particular, the metabolic syndrome is thought to be associated with platelet activation, which could drive both a prothrombotic phenotype and liver inflammation and fibrosis with the progression of MASLD towards more advanced stages of the disease [3, 8, 9, 10].

Platelets seem to be involved in the pathogenesis and progression of lower liver fibrosis stages (≤ F2) in MASLD, as they are actively recruited in the liver by Kupffer cells during these initial stages and persistently throughout the progression of the disease [11].

The role of platelets in the pathogenesis of MASLD is increasingly recognised. In fact, platelet‐derived factors contribute to inflammation, fibrotic deposition and vascular dysfunction [7, 12].

Several mechanisms have been implicated in platelet activation in patients with obesity and insulin resistance. Mediators such as thromboxane A_2_ (TXA_2_) [13, 14, 15, 16], platelet releasate and platelet‐derived microvesicles contain cytokines and growth factors, such as platelet‐derived growth factor (PDGF) and transforming growth factor‐beta (TGF‐β), which activate hepatic stellate cells (HSCs) and drive fibrosis [7, 17, 18] resulting in significant architectural distortion of the liver and promoting thrombotic complications, including portal vein thrombosis, commonly observed in cirrhotic patients [13, 19].

The potential role of platelets in the progression of MASLD ≤ F2 is highlighted by the evidence that even without clear explanation, platelet count is one of the most valuable parameters incorporated into formulas for diagnosing liver fibrosis or cirrhosis (such as Fibrosis‐4 index (FIB‐4), FAST score, AGILE 3+ and 4, among others) [20].

Understanding the role of platelets in the progression from MASLD to MASH and ultimately to liver cirrhosis is paramount for the development of targeted therapeutic approaches, aimed at arresting or even reversing the progression of the disease and possibly restoring the haemostatic balance. Using a comprehensive platelet profiling, we investigated platelet function across different MASLD stages, from ≤ F2 fibrosis to F3–F4 fibrosis, to obtain hints on the role of platelets in disease progression. Specifically, we investigated the relationship between platelets and fibrogenesis, defining the different stages of MASLD.

## 2 Methods

### 2.1 Study Design

This study was an experimental, non‐pharmacological, cross‐sectional investigation approved by the Ethics Committee of the Verona and Rovigo Provinces (Protocol 1612CESC, Amendment 2). The study adheres to the highest ethical standards and complies with the principles of the Helsinki Declaration and the Oviedo Convention, as well as relevant national and European regulations. Before enrolment, all participants were required to provide their written informed consent. Study participants were identified using unique codes, ensuring their privacy and the confidentiality of their medical data (pseudonymization).

The study population comprised MASLD patients with low fibrosis (≤ F2) and with high fibrosis (F3–F4), which were evaluated using liver stiffness measurement (LSM) obtained with transient elastography using Fibroscan (Echosense, Paris, France). Healthy individuals were also included, representing a control group for biomarker and imaging comparison.

Participants underwent a clinical examination, recording of anthropometrical parameters LSM, a comprehensive biochemical profile, and specific investigational analyses on predefined biomarkers. Non‐invasive tests, such as FIB‐4, AST‐to‐platelet ratio index (APRI) and AST‐to‐ALT ratio, were also calculated for all participants.

### 2.2 Inclusion and Exclusion Criteria

Participants in the study had to be at least 18 years old. All participants (both healthy volunteers and patients) were consecutively enrolled. For patients with MASLD, regardless of the stage of fibrosis, a recent diagnosis of liver steatosis confirmed by ultrasound or CT imaging was required, along with evidence of at least one metabolic disorder and limited alcohol consumption (less than 210 and 140 g/week, for males and females respectively) [3]. Patients with decompensated liver cirrhosis were excluded, whereas those enrolled in the F3–F4 group must have had cirrhosis of metabolic origin and specific clinical criteria [3], or at least a severe fibrosis (F3), defined using Fibroscan.

Patients were excluded if pregnant, refusing consent, using certain medications (antiplatelet agents, anticoagulants, corticosteroids or non‐steroidal anti‐inflammatory drugs), having blood disorders, active liver cancer, thrombosis or liver diseases unrelated to metabolic disorder. Smoking had to be avoided on the morning of blood and urine sample collection.

Individuals were included in the control group if healthy and not taking any drugs, whereas they were excluded in the presence of a history or signs of MASLD, cirrhosis or any other significant metabolic disorder, liver diseases or any other chronic disease.

### 2.3 Routine Clinical Evaluations

As part of routine clinical evaluations, all participants underwent a comprehensive panel of laboratory tests aimed at assessing their overall health and liver function. These included liver function tests, such as alanine aminotransferase (ALT), aspartate aminotransferase (AST) and bilirubin levels, as well as a complete blood count to evaluate general haematological status. Additionally, metabolic parameters such as fasting glucose and lipid profile were analysed to identify any underlying metabolic conditions.

Haemochromocytometric analysis including platelet count, mean platelet volume (MPV), percentage of reticulocytes (RET), immature reticulate fraction percentage (IRF) and immature platelet fraction percentage (IPF) was measured in whole blood collected in SMonovette tubes (Sarstedt) containing Sodium Citrate 3.2% and assessed using automated flow cytometry Dasit Group analyser (Sysmex XN‐9100 Haematology Analyser). Urine creatinine was measured using the enzymatic CREP2 assay on the cobas c702 (Roche, Mannheim, Germany). Estimated glomerular filtration rate was obtained by means of the CKD‐EPI formula, 2021 updated version.

### 2.4 Fibroscan

Every patient and healthy individual was examined with vibration controlled transient elastography (VCTE) using Fibroscan (Echosense, Paris, France), a non‐invasive diagnostic tool used to assess LSM, which correlates with the degree of liver fibrosis or scarring [3, 5, 20, 21].

To further stratify our study population based on LSM values, we applied a cutoff of 10 kPa. Patients with LSM ≤ 10 kPa were classified as having mild to moderate fibrosis (≤ F2), whereas those with LSM > 10 kPa were considered to have severe fibrosis or cirrhosis (F3–F4). This threshold was selected based on the “rule of 5” from the Baveno VII guidelines, which recommend suspecting Compensated Advanced Chronic Liver Disease when LSM exceeds 10 kPa [22]. We also evaluated an alternative cut‐off of 8 kPa, as per EASL–EASD–EASO Clinical Practice Guidelines on MASLD [3].

### 2.5 Blood Sample Collection

A clean puncture of an antecubital vein was performed with a 19‐gauge needle (Safety‐Multifly‐Set, Sarstedt, Nümbrecht, Germany) and blood collection was performed without applying venostasis. After discarding the first 2–3 mL of blood, SMonovette tubes (Sarstedt) containing trisodium citrate 3.2% were used as collection tubes and anticoagulant was immediately mixed with blood by gentle inversion. Platelet‐rich plasma (PRP) was obtained by centrifugation of blood at 180 g at room temperature for 15 min, whereas platelet‐poor plasma (PPP) was obtained by centrifugation of 1 mL of PRP at 1600 *g* for 5 min, in either case without using the brake.

### 2.6 Platelet Rich Plasma Preparation

Platelet Rich Plasma (PRP) samples obtained from blood centrifugation were diluted using the PPP of the same subject to reach the equal concentration among all subjects in the same experiment. In microfluidic perfusion, the defined platelet concentration in PRP samples was 100 × 10^3^ platelets/μL when it was possible; otherwise, the maximal common platelet concentration reachable from all subjects was used. In FACS (fluorescence‐activated cell sorting) analysis, the platelet concentration in PRP samples was set to 20 × 10^3^ platelets/μL.

### 2.7 Assay of Urinary 11‐Dheydro‐Tromboxane B2

Overnight urine samples were collected and stored at −80°C. Samples were thawed and assayed for 11‐dehydro‐Tromboxane B2 (11‐dh‐TXB_2_) using a commercially available enzyme‐linked immunoassay (ELISA) (Cayman Chemical, Ann Arbor, Michigan, USA). Urinary 11‐dh‐TXB_2_, a stable metabolite reflecting systemic TXA2 production, was normalised to urinary creatinine concentration and expressed as pg. of 11‐dh‐TXB_2_ per mg of creatinine.

### 2.8 Assay of Plasma Soluble P‐Selectin

Plasma samples, obtained from blood collected using 3.2% sodium citrate as anticoagulant and stored at‐80°C, were thawed and assayed for plasma soluble P‐selectin, with a commercially available enzyme‐linked immunoassay (ELISA) kit (Abcam, Cambridge, United Kingdom). The measurements of plasmatic soluble P‐selectin are reported as ng/mL.

### 2.9 Flow Cytometry Analysis of Platelets

Annexin V expression was analysed in PRP by flow cytometry as previously described [23]. PRP was diluted to 20,000 platelets/μL in binding buffer (Invitrogen by Thermo Fisher Scientific, Bender MedSystems GmbH, Vienna, Austria). PRP (10 μL) was incubated with FITC‐Annexin V (5 μL), which specifically binds to the platelet‐exposed phosphatidylserine (PS), with or without the platelet agonist TRAP‐6 20 μmol/L, a peptide fragment of the “tethered ligand” receptor for thrombin. After 15 min of incubation at room temperature, in the dark, the reaction was stopped by adding BD Pharmingen Annexin V Binding Buffer 1X.

Incubation with PE‐CD62p (5 μL), a platelet activation marker, with or without TRAP‐6 20 μmol/L was performed. After 15 min at room temperature in the dark, the reaction was stopped by adding Hepes Buffer 1X. Using FICT‐PAC‐1 (5 μL), the corresponding antibody used to investigate the active form of integrin αIIbβ3 complex, the Fibrinogen receptor was explored, with or without TRAP‐6 20 μmol/L. The incubation was performed always in the dark at room temperature and the reaction was stopped by adding Hepes Buffer 1X.

For the analysis of hetero‐aggregates between platelets and monocytes (APC‐CD41a/FITC‐CD14) or between platelets and neutrophils (APC‐CD41a/PE‐CD66), 15 μL of whole blood was incubated for 20 min at room temperature in the dark with 5 μL of each specific antibody, with or without TRAP‐6 20 μmol/L. The reaction was stopped by adding BD FACS Lysing Solution 1X, which induces the lysis of red blood cells and, due to the formaldehyde concentrated between 1% and 2%, fixes the sample.

The fluorescently labelled antibodies FITC‐Annexin V (51‐65874×), PE‐CD62p (550561, clone AC1.2), FITC‐PAC‐1 (340507, clone PAC‐1), APC‐CD41a (559777, clone HIP8), FITC‐CD14 (555397, clone M5E2) and PE‐CD66 (551480, clone B1.1/CD66) were purchased from Becton‐Dickinson Company BD Biosciences (San Jose, CA, USA).

Flow cytometry analysis was performed using BD FACS CANTO II, and data were analysed using BD FACSDiva Software (BD Biosciences).

### 2.10 Assay of Platelet Adhesion

Platelet adhesion under flow was performed using a Microfluidic Cellix platform (Cellix Ltd., Ireland) composed of a pump (Mirus Evo nano pump), an optic microscope (Leica DM IRB, objective magnification 20×, numerical aperture 0.30) equipped with an EXi Blue fluorescence microscopy camera (1392 × 1040 pixels, 800 Mb/s bandwidth capacity, 15 frames per seconds full resolution at 14 bits, 30 MHz, EXi Blue Q IMAGING) and Vena8 Fluoro+ biochips (Cellix Ltd). Channels of the biochips were coated with 200 μg/mL equine tendon collagen (Horm collagen, Takeda) overnight at 4°C in a humidified box (Philipose et al. 2010). To avoid unspecific binding, the channels were blocked with 10 μg/mL bovine serum albumin (BSA, Sigma Aldrich) for 30 min at room temperature, followed by washing steps with saline solution (NaCl 9 g/L). PRP was diluted with physiological saline solution to obtain a final concentration of 1.10^8^/mL platelets. Each specimen was then fluxed through the channels with a shear stress of 10 dyn for 3 min. During the experiment, the temperature of the multichannel Biochip Vena8 Fluoro+ was kept at 37°C. After the perfusion, 20 images for each experiment were taken along the whole channels and analysed with Duco Cell software (Cellix Ltd., Dublin, Ireland) which provides cell counting and analysis of morphological parameters of cells including area, diameter, perimeter, ellipticity and form‐factor. Platelet adhesion on the collagen surface was calculated and expressed as areas occupied by cell aggregates (Arbitrary Units, AU).

### 2.11 Statistical Analysis

The sample size was calculated a priori, with the primary endpoint being the difference in plasma soluble P‐selectin (sP‐selectin) concentration compared to control individuals. Based on previous literature [24], the expected difference (Δ) between groups was set at 40% of the mean. The significance level (*α*) was set at 0.05, corresponding to *Z*_*α*/2_≈1.96, and the desired power was 80%, corresponding to *Z*_*β*_≈0.84.

A minimum sample size of 18 subjects per group was calculated to detect a 40% difference in sP‐selectin concentration with 80% power and a 5% significance level.

For inferential statistics, non‐parametric tests were applied according to the results of the analysis of data distribution performed with the Kolmogorov–Smirnov test and the D'Agostino and Pearson test. The Kruskal‐Wallis test was applied for multiple comparisons (namely healthy subjects, patients with MASLD ≤ F2, and F3–F4) of continuous variables, followed by the Dwass‐Steel‐Critchlow‐Fligner as post hoc test for pairwise comparisons.

For the comparison of categorical variables, the Friedman test and the Pearson's Chi‐square test were used.

Data are expressed in figures and tables as individual data and median with 95% confidence intervals (CI) in the main text. *p* value < 0.05 was set as statistically significant.

Statistical analyses were conducted using the statistics software SPSS, Version 20 (IBM Corp. Released 2011. IBM SPSS Statistics for Windows, Version 20.0. Armonk, NY: IBM Corp.) and the statistical software GraphPad Prism version 10.0.0 for Windows (GraphPad Software, San Diego, California USA).

## 3 Results

A total number of 192 subjects were enrolled in this cross‐sectional case–control study: 62 control subjects, 82 MASLD ≤ F2 patients and 48 patients with MASLD F3–F4.

Healthy subjects were the reference for experimental tests and differ from patients with chronic liver disease as for indices of metabolic disorder and liver disease, as expected, but also for age and sex (Table 1).

**TABLE 1:** Descriptive statistics for the total population enrolled and the three groups of subjects participating in the study.

Variable	Total population (*N* = 192)	Healthy controls (*N* = 62)	MASLD ≤ F2 (*N* = 82)	MALSD F3–F4 (*N* = 48)	*p*	Pairwise comparison *p* ^†^		
						Controls versus MASLD ≤ F2	Controls versus MASLD F3–F4	MASLD ≤ F2 versus MASLD F3–F4
*Demographic and anthropometric parameters*								
Sex, male	124 (64.6)	32 (51.6)	26 (31.7)	10 (20.8)	**< 0.01** *	**< 0.01**	**< 0.001**	**< 0.01**
Age, years	51.91 ± 14.8	38.0 (29.0–50.3)	56.0 (47.5–60.0)	64.5 (55.8–71.0)	**< 0.001** ^**§**^	**< 0.001**	**< 0.001**	**< 0.001**
BMI, Kg/m^2^	26.4 ± 4.4	22.5 (21.5–24.9)	27.2 (24.4–29.2)	25.2 (23.0–31.3)	**< 0.01** ^**§**^	**< 0.001**	**< 0.01**	0.76
Obesity	28 (14.6)	1 (1.6)	15 (18.3)	12 (25.0)	**0.02** *	**< 0.05**	**0.01**	0.58
*Comorbidities and habits*								
Hypertension	62 (32.3)	3 (4.8)	37 (45.1)	22 (45.8)	**< 0.001** *	**< 0.001**	**< 0.001**	0.97
Hyperlipidaemia	64 (33.3)	1 (1.6)	49 (59.8)	14 (29.2)	**< 0.001** *	**< 0.001**	**< 0.001**	**< 0.001**
Type 2 diabetes mellitus	29 (15.1)	0 (0.0)	7 (8.5)	22 (45.8)	**< 0.001** *	0.06	**< 0.001**	**< 0.001**
IFGT (pre‐diabetes)	19 (9.9)	0 (0.0)	10 (12.2)	9 (18.8)	**< 0.01** *	**0.02**	**< 0.01**	0.57
Metabolic syndrome	32 (16.7)	0 (0.0)	19 (23.2)	13 (27.1)	**< 0.001** *	**< 0.001**	**< 0.001**	0.84
CVD history	7 (3.6)	0 (0.0)	1 (1.2)	6 (12.5)	**< 0.01** *	0.50	**0.02**	0.17
ACS	2 (1.0)	—	—	2 (4.2)				
Stent or bypass	1 (0.5)	—	—	1 (2.1)				
Stroke	1 (0.5)	—	—	1 (2.1)				
Arrhythmias	1 (0.5)	—	—	1 (2.1)				
Others	2 (1.0)	—	1 (1.2)	1 (2.1)				
*Blood and urine laboratory tests*								
WBC, x109/L	5.9 ± 2.4	5.7 (4.7–6.8)	6.1 (5.4–7.1)	4.3 (3.1–5.9)	**< 0.001** ^**§**^	0.46	**< 0.01**	**< 0.001**
RBC, x1012/L	4.5 ± 1.1	4.6 (4.1–5.0)	4.9 (4.6–5.4)	3.2 (2.6–3.8)	**< 0.001** ^**§**^	**0.02**	**< 0.001**	**< 0.001**
Hb, g/L	135.4 ± 29.4	137.0 (123.5–148.0)	148.0 (135.3–159.0)	104.5 (85.0–120.5)	**< 0.001** ^**§**^	**< 0.01**	**< 0.001**	**< 0.001**
HCT, L/L	0.4 ± 0.1	0.4 (0.3–0.4)	0.4 (0.4–0.5)	0.3 (0.3–0.4)	**< 0.001** ^**§**^	**0.02**	**< 0.001**	**< 0.001**
MCV, fL	89.9 ± 6.9	88.7 (86.1–91.6)	88.6 (85.1–91.5)	95.3 (90.7–100.8)	**< 0.001** ^**§**^	> 0.99	**< 0.001**	**< 0.001**
PLT (in sodium citrate), x109/L	197.3 ± 84.1	233 (197.5–268.0)	226.5 (205.3–270.8)	70.5 (50.8–96.0)	**< 0.001** ^**§**^	> 0.99	**< 0.001**	**< 0.001**
PDW (in sodium citrate), %	12.0 ± 2.3	11.3 (9.9–13.5)	12.1 (10.8–13.4)	11.4 (9.9–13.4)	0.26^§^	0.70	> 0.99	0.40
MPV (in sodium citrate), fL	10.3 ± 1.1	10.1 (9.3–14.3)	10.4 (9.7–10.8)	10–2 (9.6–10.9)	0.75^§^	> 0.99	> 0.99	> 0.99
NEU, x109/L	3.4 ± 2.0	3.1 (2.4–4.1)	3.5 (2.7–4.2)	2.5 (1.8–3.5)	**< 0.01** ^**§**^	0.88	0.07	**< 0.01**
LYMPH, x109/L	1.7 ± 0.8	1.8 (1.5–2.1)	1.9 (1.6–2.2)	1.0 (0.6–1.4)	**< 0.001** ^**§**^	> 0.99	**< 0.001**	**< 0.001**
MONO, x109/L	0.5 ± 0.3	0.5 (0.4–0.6)	0.5 (0.4–0.6)	0.4 (0.3–0.6)	0.24^§^	> 0.99	> 0.99	0.27
RET, %	1.3 (1.0–1.8)	1.1 (0.9–1.3)	1.3 (1.1–1.6)	2.0 (1.7–2.5)	**< 0.001** ^**§**^	**< 0.01**	**< 0.001**	**< 0.001**
IRF, %	9.3 (7.0–12.6)	7.8 (5.9–9.7)	9.2 (7.1–11.6)	11.9 (9.3–15.6)	**< 0.001** ^**§**^	0.24	**< 0.001**	**< 0.01**
IPF (in sodium citrate), %	3.6 (2.4–5.4)	4.1 (2.4–5.5)	3.2 (2.3–4.7)	3.7 (2.6–5.8)	0.20^§^	0.26	> 0.99	0.61
AST, IU/L	28.0 (22.0–43.0)	22.0 (18.0–28.0)	28.0 (22.0–42.0)	38.0 (28.0–72.0)	**< 0.001** ^**§**^	**< 0.001**	**< 0.001**	**< 0.001**
ALT, IU/L	30.0 (21.0–47.5)	21.0 (14.0–30.5)	34.0 (22.8–56.3)	32.0 (23.0–48.5)	**< 0.001** ^**§**^	**< 0.001**	**< 0.01**	0.74
GGT, IU/L	33.0 (17.0–69.0)	13.5 (10.0–24.8)	29.0 (18.0–67.0)	65.0 (41.8–136.8)	**< 0.001** ^**§**^	**< 0.001**	**< 0.001**	**< 0.001**
ALP, IU/L	94.0 (68.0–120.0)	NA	71.0 (61.0–94.0)	116.0 (91.5–160.5)	**< 0.001** ^**§**^	0.61	0.10	**< 0.001**
Tot. bilirubin, mg/dL	0.7 (0.5–1.6)	0.5 (0.3–0.6)	0.6 (0.5–0.8)	1.6 (0.8–3.0)	**< 0.001** ^**§**^	0.17	**< 0.001**	**< 0.001**
Ferritin, ug/L	184.0 (71.9–348.0)	47.0 (29.3–97.3)	130.0 (59.0–252.5)	330.3 (193.3–720.5)	**< 0.001** ^**§**^	0.08	**< 0.01**	**< 0.001**
Creatinine, mg/dL	0.8 (0.7–1.0)	0.9 (0.7–1.0)	0.8 (0.7–1.0)	0.8 (0.7–1.1)	0.90^§^	> 0.99	> 0.99	> 0.99
Albumin, g/L	40.2 ± 7.2	48.0 (44.7–54.9)	45.3 (40.1–47.0)	34.9 (32.7–40.1)	**< 0.001** ^**§**^	0.23	**< 0.001**	**< 0.001**
Total cholesterol, mg/dL	175.0 ± 44.7	179.0 (165.3–203.0)	188.5 (162.5–211.4)	127.0 (94.5–177.5)	**< 0.001** ^**§**^	0.89	**< 0.001**	**< 0.001**
LDL‐cholesterol, mg/dL	106.7 ± 34.7	108.0 (87.0–125.0)	115.7 (92.6–135.0)	58.5 (41.9–101.1)	**< 0.001** ^**§**^	0.39	**< 0.01**	**< 0.001**
HDL‐cholesterol, mg/dL	53.1 ± 14.5	57.5 (52.0–71.0)	50.0 (42.0–58.0)	49.0 (38.5–60.5)	**< 0.001** ^**§**^	**0.01**	0.07	0.99
Triglycerides, mmol/L	107.1 ± 47.3	83.0 (67.0–99.0)	117.7 (74.6–152.5)	97.0 (66.0–126.0)	**< 0.001** ^**§**^	**< 0.01**	0.59	0.13
Glycaemia, mg/dL	102.0 ± 31.8	88.0 (82.0–93.0)	95.0 (88.3–102.0)	106.0 (88.0–147.0)	**< 0.001** ^**§**^	**< 0.001**	**< 0.001**	**0.03**
Urinary 11‐dh‐TXB_2_/creatinine ratio, ng/g	2755 (1756–6392)	2343 (1688–3211)	1908 (857–3258)	9581 (7125–13 628)	**< 0.001** ^**§**^	> 0.99	**< 0.001**	**< 0.001**
Plasmatic sP‐selectin, ng/mL	136.5 (86.7–250.0)	79.8 (62.4–110.2)	136.5 (107.3–175.8)	309.5 (263.8–356.0)	**< 0.001** ^**§**^	**0.001**	**< 0.001**	**< 0.001**
*Non‐invasive tests and non‐invasive assessments*								
FIB‐4	1.14 (0.78–2.58)	0.73 (0.55–0.96)	1.03 (0.78–1.38)	6.29 (3.81–8.74)	**< 0.001** ^**§**^	**< 0.01**	**< 0.001**	**< 0.001**
AST to ALT ratio	1.08 ± 0.45	1.00 (0.86–1.25)	0.95 (0.65–1.00)	1.23 (1.11–1.52)	**< 0.001** ^**§**^	**< 0.01**	**< 0.01**	**< 0.001**
APRI	0.31 (0.22–0.65)	0.22 (0.18–0.29)	0.29 (0.23–0.41)	1.35 (0.63–2.17)	**< 0.001** ^**§**^	**< 0.01**	**< 0.001**	**< 0.001**
CAP, dB/m	229.5 (179.8–289.5)	180.5 (157.0–218.5)	280.0 (238.0–324.0)	249.0 (204.5–294.5)	**< 0.001** ^**§**^	**< 0.001**	**0.04**	0.27
LSM, kPa	5.5 (4.1–9.1)	4.1 (3.6–4.6)	5.9 (4.5–7.3)	16.3 (12.4–23.6)	**< 0.001** ^**§**^	**< 0.001**	**< 0.001**	**< 0.001**
Spleen size, cm	12.1 ± 3.5	9.4 ± 0.6	10.2 ± 1.4	14.2 ± 3.8	**< 0.001** ^**§**^	0.18	**< 0.01**	**< 0.001**

*Note:* Numbers in bold represent statistical significance.

Abbreviations: 11‐dh‐TXB_2_, urinary 11‐dehydrothromboxane B_2_; ACS, acute coronary syndrome; ALP, alkaline phosphatase; ALT, alanine aminotransferase; APRI, AST to platelet ratio index; AST aspartate aminotransferase; BMI, body mass index; CAP, controlled attenuation parameter; CVD, cardiovascular disease; FIB‐4, Fibrosis‐4 index; GGT, gamma‐glutamyl transferase; Hb, haemoglobin; HCT, haematocrit; HDL, high density lipoprotein; IFGT, impaired fasting glucose tolerance; IPF, immature platelet fraction in the platelet count; IRF, immature reticulate fraction in the reticulocytes count; LDL, low density lipoprotein; LSM, liver stiffness measurement; LYMPH, lymphocytes; MASLD ≤ F2, metabolic dysfunction‐associated steatotic liver disease with low liver fibrosis; MASLD F3–F4, metabolic dysfunction‐associated steatotic liver disease with advanced liver fibrosis/cirrhosis; MCV, mean corpuscular volume; MONO, monocytes; MPV, mean platelet volume; NEU, neutrophils; PDW, platelet distribution width; PLT, platelet count; RBC, red blood cell count; RET, reticulocytes; WBC, white blood cell count.

* Pearson's Chi‐square.

^§^ Kruskal‐Wallis' test.

^†^ Dwass‐Steel‐Critchlow‐Fligner.

Comparison of clinical data among patient groups showed that obesity was more frequently observed in the MASLD ≤ F2 and F3–F4 groups compared to the control group, with no significant difference between MASLD ≤ F2 and F3–F4. Similarly, DM was significantly more prevalent in the cirrhosis group compared to both the control and MASLD ≤ F2 groups.

Consistent with the diagnosis of advanced fibrosis/liver cirrhosis were the alterations in biochemical profile (plasma albumin, liver enzymes, total and LDL cholesterol) and the results of haemochromocytometric analysis, showing a trilinear cytopenia, with mean platelet count in the range of moderate thrombocytopenia (median difference between healthy subjects and cirrhotic patients: −162.5 × 10^3^/μL, 95% CI −173.0 to −136.0; between ≤ F2 and F3–F4 patients: −156.0 × 10^3^/μL, 95% CI −174.0 to −138.0) (see Figure S1, Panel A). The analysis on the Immature Platelet Fraction (IPF) to address a plausible disparity among healthy control, ≤ F2 and F3–F4 patients did not show any statistical difference (see Figure S1, Panel B).

Non‐invasive liver fibrosis tests (such as NFS, FIB‐4 and LSM) revealed significant differences between groups, with the highest values observed in the F3–F4 fibrosis group (*p* < 0.001, for all comparisons, see also Table 1).

### 3.1 Biomarkers of In Vivo Platelet Activation

To assess in vivo platelet activation, we measured the urinary excretion of 11‐dh‐TXB_2_, a stable metabolite of TXA2, and expressed the results as a function of urinary creatinine.

A statistically significant difference among the groups was observed (*p* < 0.001), with the highest concentrations found in the F3–F4 group.

The median difference in 11‐dh‐TXB_2_ levels between MASLD F3–F4 patients and healthy controls was 6931.00 ng/g creatinine (95% CI: 5290.00–8363.00).

**FIGURE 1:**
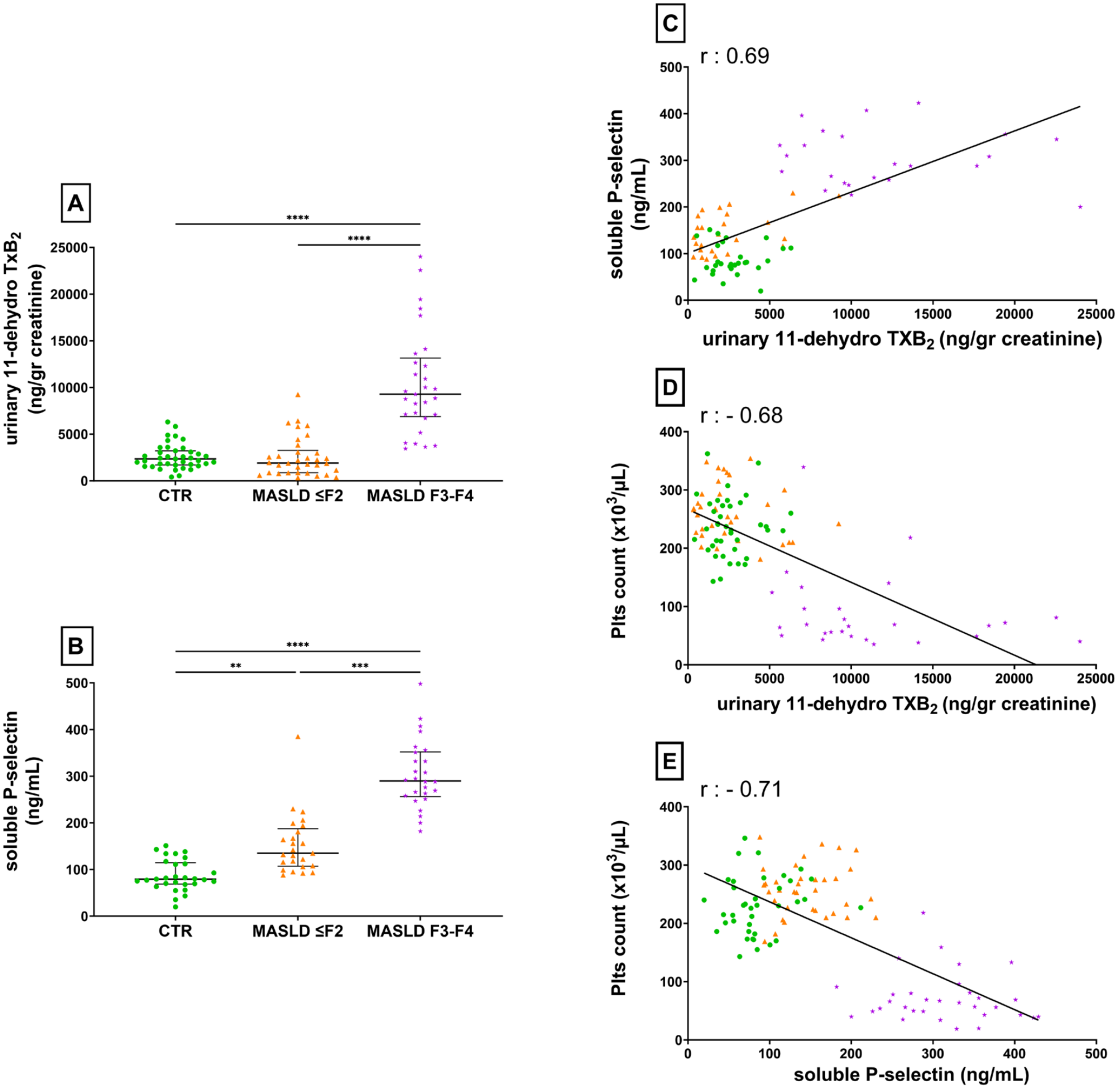
Distribution of platelet activation markers and their correlations with platelet count among control, MASLD ≤ F2 and F3‐F4 groups. Panels (A) and (B) show the distributions of urinary 11‐dehydro Thromboxane B2 and soluble P‐selectin. (A) Urinary 11‐dehydro Thromboxane B2 levels (ng/g creatinine) in the three groups: healthy controls (CTR), patients with MASLD ≤ F2 and patients with F3–F4 fibrosis (MASLD F3–F4). (B) Plasma soluble P‐selectin (sP‐selectin) levels (ng/mL) among the three groups: CTR, MASLD ≤ F2 and MASLD F3–F4. Panels (C), (D) and (E) show correlations between platelet activation markers and platelet count. (C) urinary 11‐dehydro Thromboxane B_2_ (ng/g creatinine) compared to plasmatic soluble P‐selectin (ng/mL): Healthy subjects (green dots), patients with MASLD ≤ F2 (orange dots) and patients with F3–F4 fibrosis (purple dots); simple linear regression representing a Pearson correlation *r* = 0.6918. (D) urinary 11‐dehydro Thromboxane B_2_ (ng/g creatinine) compared to platelets count (×10^3^/μL): healthy subjects (green dots), patients with MASLD ≤ F2 (orange dots) and patients with F3–F4 fibrosis (purple dots); simple linear regression representing a Pearson correlation *r* = −0.6813. (E) plasmatic soluble P‐selectin (ng/mL) compared to platelets count (x10^3^/μL), healthy subjects (green dots), patients with MASLD ≤ F2 (orange dots) and patients with F3–F4 fibrosis (purple dots); simple linear regression representing a Pearson correlation *r* = −0.7127. **Kruskal‐Wallis test, *p*‐value < 0.01; ***Kruskal‐Wallis test, *p*‐value < 0.0001; ****Kruskal‐Wallis test, *p*‐value < 0.0001; MASLD F3–F4, patients with advanced fibrosis/cirrhosis group; CTR, control group; MASLD ≤ F2, Metabolic dysfunction‐Associated Steatotic Liver Disease with low liver fibrosis; MASLD F3–F4, Metabolic dysfunction‐Associated Steatotic Liver Disease with advanced liver fibrosis/cirrhosis; Plts, platelets.

The median difference between MASLD F3–F4 and MASLD F1–F2 patients was 7365.00 ng/g creatinine (95% CI: 5257.00–8764.00) (see also Figure 1, panel A).

As for plasma sP‐selectin, which is mostly shed from activated platelets, F3–F4 group patients displayed the highest median plasma concentration, significantly different from that in the other two groups (median difference between control and cirrhosis groups is 210.70 ng/mL, 95% CI: 181.30–238.20; median difference between ≤ F2 and F3–F4 patients groups is 155.00 ng/mL, 95% CI: 116.00 to 183.00) (see also Figure 1, panel B). Unlike urinary 11‐dh‐TXB_2_, plasma sP‐selectin was higher in patients with MASLD ≤ F2 compared to healthy controls (the median difference is 55.70 ng/mL, 95% CI: 33.50–81.60).

### 3.2 Relationship Between Biomarkers of In Vivo Platelet Activation and Platelet Count

We explored plausible relations between the two biomarkers, urinary 11‐dh‐TXB_2_ and plasmatic sP‐selectin. Linear correlation was statistically significant in the cumulated analysis of the three groups (see Figure 1, panel C).

A significant correlation was observed between the two markers of in vivo platelet activation and platelet count. In either case, the lower the platelet count, the higher the concentration of urinary 11‐dh‐TXB_2_ (see Figure 1, panel D) and plasma sP‐selectin (see Figure 1, panel E). It is possible to appreciate the distinction once again between F3–F4 patients and healthy or MASLD ≤ F2 subjects according to a negative coefficient linear regression correlation (*r* in 11‐dh‐TXB_2_: −0.68; *r* in sP‐selectin: −0.71).

### 3.3 Relationship Between Biomarkers of In Vivo Platelet Activation and Indices of Liver Fibrosis

**FIGURE 2:**
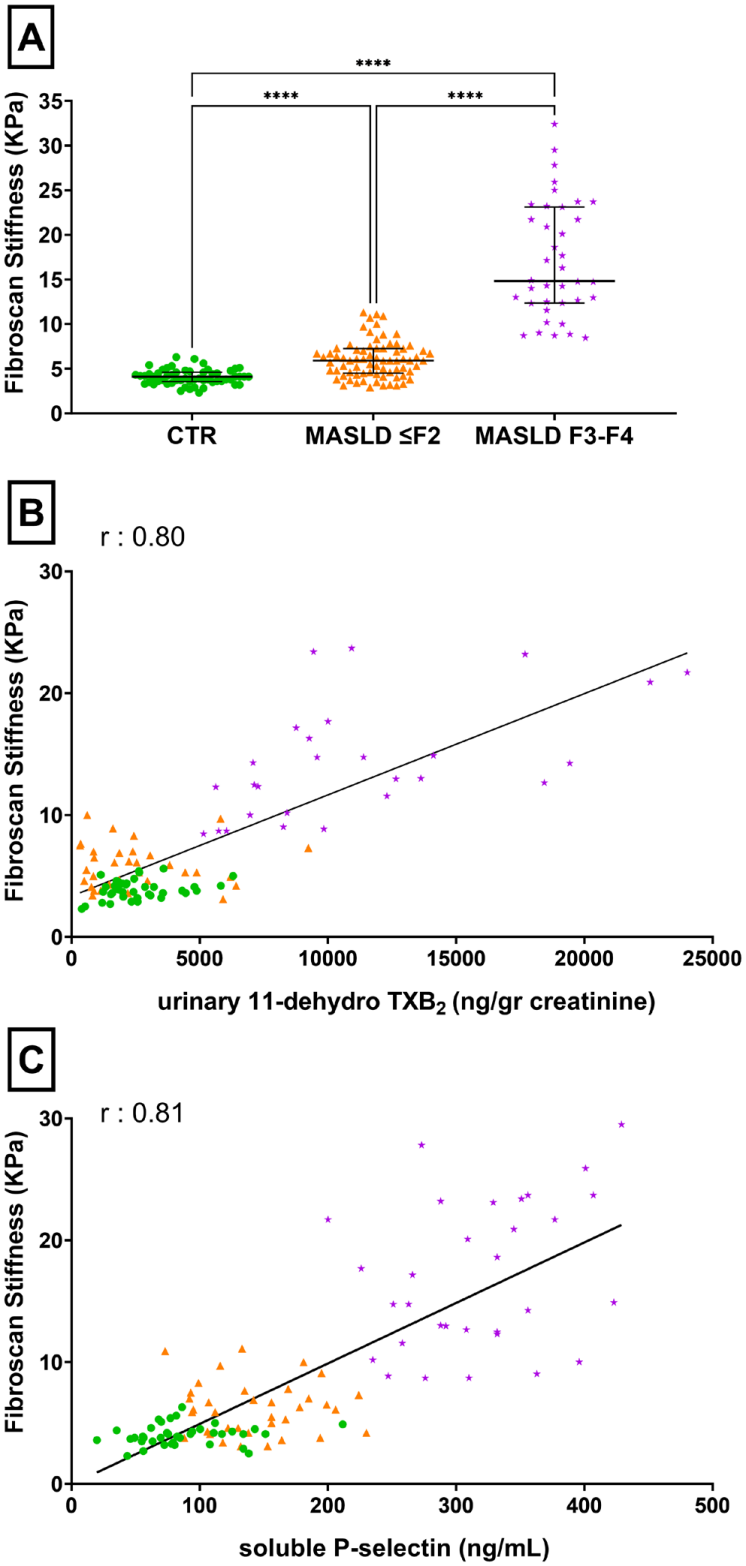
Distribution of liver stiffness measured with Fibroscan and its correlations with platelet activation markers, across control, MASLD ≤ F2 and F3–F4 groups. (A) Liver stiffness (kPa) using Fibroscan among the three groups: control group (CTR), MASLD ≤ F2 group and F3–F4 fibrosis group. Panels (B) and (C) show correlations between platelet activation markers and liver stiffness. (B) Correlation of urinary 11‐dehydro Thromboxane B_2_ (ng/g creatinine) with liver stiffness (kPa): healthy subjects (green dots), patients with MASLD ≤ F2 (orange dots) and patients with F3–F4 fibrosis (purple dots); simple linear regression representing a Pearson correlation *r* = 0.7990. (C) Correlation of plasmatic soluble P‐selectin (ng/mL) with liver stiffness (kPa): healthy subjects (green dots), patients with MASLD ≤ F2 (orange dots) and patients with F3–F4 fibrosis (purple dots); simple linear regression representing a Pearson correlation *r* = −0.8015. ****Kruskal‐Wallis test, *p*‐value < 0.0001; MASLD F3–F4, patients with advanced fibrosis/cirrhosis group; CTR, control group; MASLD ≤ F2, Metabolic dysfunction‐Associated Steatotic Liver Disease with low liver fibrosis; MASLD F3–F4, Metabolic dysfunction‐Associated Steatotic Liver Disease with advanced liver fibrosis/cirrhosis.

Liver fibrosis, investigated via liver stiffness measurement (LSM, expressed in kPa), using Fibroscan, helps to differentiate the three groups according to absence or severity of the disease. Statistically significant differences among all three populations were observed (p value < 0.001, see also Table 1). The median difference between healthy subjects and MASLD ≤ F2 was 1.8 kPa (95% CI: 1.2–2.3); the median difference between control and F3–F4 groups was 10.7 kPa (95% CI: 9.6–14.8); the median difference between MASLD ≤ F2 and F3–F4 group was 8.92 kPa (95% CI: 8.0–12.5) (see Figure 2, Panel A).

Given the observed differences in urinary 11‐dh‐TXB_2_, we also found a statistically significant correlation with fibrosis (see Figure 2, Panel B). Similarly, a significant correlation was observed, also considering plasmatic sP‐selectin (see Figure 2, Panel C), either considering the three groups of subjects or the F3–F4 patients alone.

### 3.4 Platelet Phenotype and Functionality in ≤ F2 and F3–F4 Groups

In the flow cytometry analyses, various platelet markers were evaluated, including binding of annexin V, P‐selectin, the active form of the αIIbβ3 complex (the integrin responsible for fibrinogen binding) and the presence of platelet‐leukocyte heteroaggregates. These experiments were conducted using both resting and TRAP‐6 (20 μM) stimulated platelets, with TRAP‐6 serving as a thrombin receptor‐activating peptide. TRAP‐6, being a strong agonist, induced significant platelet activation, not including blood coagulation, across all flow cytometry studies.

**FIGURE 3:**
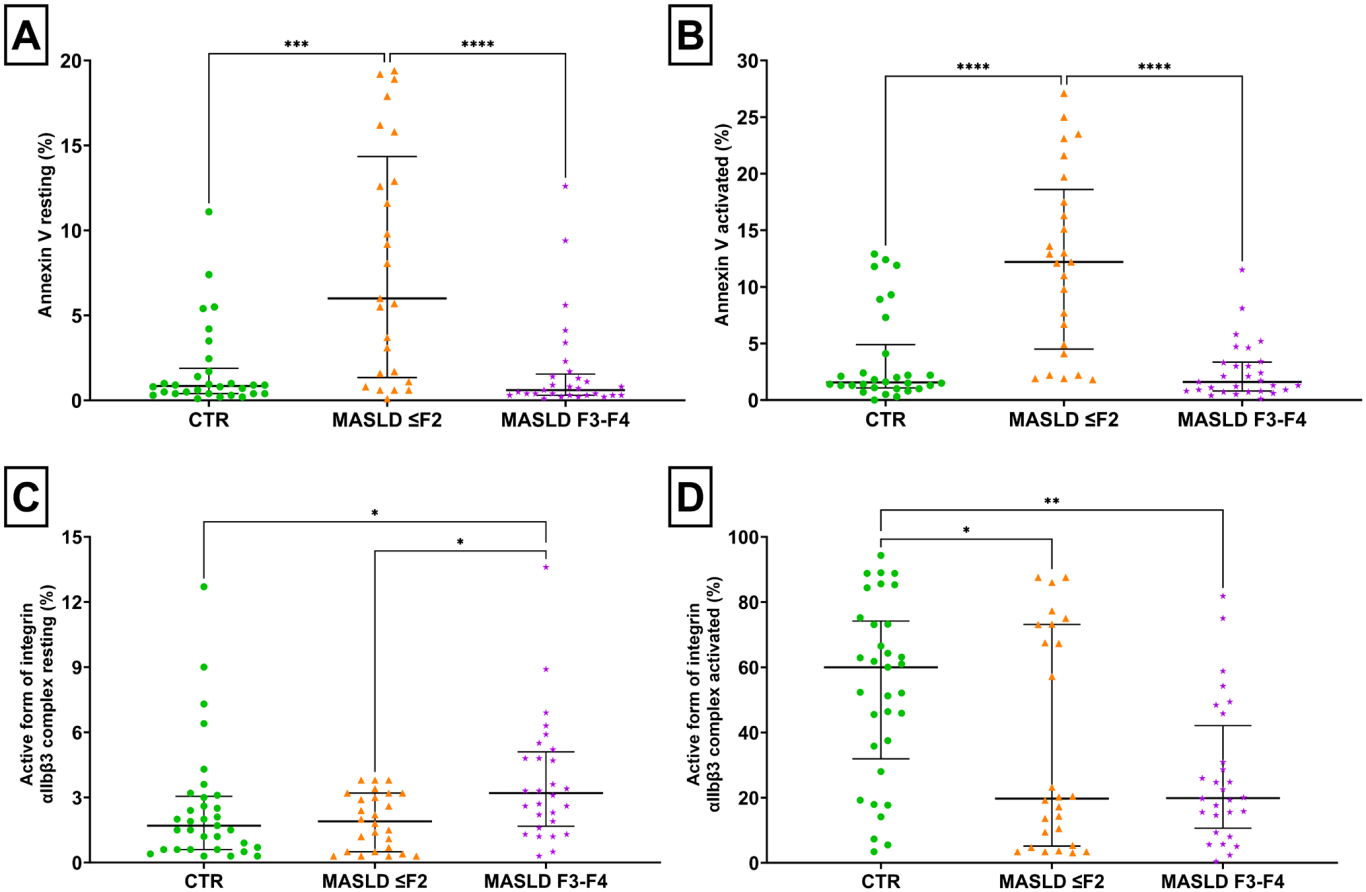
(A) Annexin V expression (%) considering platelets in resting condition among the three groups: control group (CTR), MASLD ≤ F2 group, and F3–F4 fibrosis group. (B) Annexin V expression (%) considering platelets in activated condition, among the three group: CTR, MASLD ≤ F2, and F3–F4 fibrosis. TRAP‐6 20 μM was used as agonist. (C) Active form of integrin αIIbβ3 complex expression (%) considering platelets in resting condition, among the three group: CTR, MASLD ≤ F2, and MASLD F3–F4 group. (D) Active form of integrin αIIbβ3 complex expression (%) considering platelets in activated condition, among the three group: CTR, MASLD ≤ F2, and MASLD F3–F4 group. TRAP‐6 20 μM was used as agonist. *Kruskal‐Wallis test, *p*‐value < 0.05; **Kruskal‐Wallis test, *p*‐value < 0.01; ***Kruskal‐Wallis test, *p*‐value < 0.0001; ****Kruskal‐Wallis test, *p*‐value < 0.0001; MASLD F3–F4, patients with advanced fibrosis/cirrhosis group; CTR, control group; MASLD ≤ F2, Metabolic dysfunction‐Associated Steatotic Liver Disease with low liver fibrosis; MASLD F3–F4, Metabolic dysfunction‐Associated Steatotic Liver Disease with advanced liver fibrosis/cirrhosis.

Annexin V‐positive resting platelets (see Figure 3, Panel A) were significantly elevated in ≤ F2 patients compared to controls (median difference: 5.15; 95% CI: 1.29 to 9.10) and F3–F4 patients (median difference: −5.40; 95% CI: −9.40 to −1.39).

After stimulation with TRAP‐6 (see Figure 3, Panel B) the percentage of Annexin V‐positive platelets increased in the three groups, with the highest percentage observed in ≤ F2 patients compared to control (median difference 10.65 percentage, 95% CI: 3.60 to 12.00) and F3–F4 groups (median difference −9.90 percentage and 95% CI: −12.50 to −5.40).

P‐selectin is an adhesion molecule contained in the alpha granules of resting platelets, which is expressed on the surface of activated circulating platelets and is highly expressed after ex vivo stimulation with platelet agonists. No statistical difference was observed among the three different groups in P‐selectin expression in resting platelets (see Figure S2, Panel A). In TRAP‐6 stimulated platelets (see Figure S2, Panel B), a statistically significant difference was observed between F3–F4 patients (*p* < 0.05) and healthy subjects (median difference: −14.50%, 95% CI: −16.50 to −2.70).

A low percentage of circulating platelets express on their surface the active form of the αIIbβ3 complex, the integrin binding fibrinogen, necessarily required for platelet aggregation. Under resting conditions (see Figure S2, Panel C) a statistical difference was present among the F3–F4 group in which the expression was higher compared to the healthy subjects group (the median difference is 1.50%, 95% CI: 0.30–2.30) with a *p* = 0.03, and ≤ F2 patients (the median difference is 1.30%, 95% CI: 0.40–2.40) with a *p* = 0.03.

When the three groups were compared using PRP stimulated with TRAP‐6, a higher response to the agonist was observed in the group of healthy subjects, compared to the MASLD ≤ F2 group (median difference −40.30%, 95% CI −41.60 to −0.10) with a *p* < 0.05, and F3–F4 patients (median difference −40.20%, 95% CI −43.20 to −13.60, *p* < 0.01) (see Figure S2, Panel D).

### 3.5 Investigation on Platelet‐Leukocyte Interaction

Circulating platelets bind to monocytes and neutrophils to form heteroaggregates, mostly through the interaction of platelet P‐selectin with counterreceptors on leukocytes (see also Figure S3, Panels A–D). Stimulation of diluted blood with TRAP‐6 increased the number of heteroaggregates. Both under resting conditions and after stimulation, the number of platelet‐monocyte complexes was lower in the group of patients with F3–F4. In particular, in resting conditions, it was observed a median difference of −8.30% (95% CI: −15.87 to −2.30) between healthy controls and F3–F4the F3–F4 patient group, and −11.60% (95% CI: −14.70 to −4.30) between MASLD ≤ F2 and F3–F4 groups. After activating stimulus with TRAP‐6, a median difference of −18.80% (95% CI: −26.30 to −4.30) was observed between healthy and F3–F4 groups, and −21.10% (95% CI −26.00 to −3.00) between MASLD ≤ F2 and F3–F4 patients. The number of platelet‐granulocytes (neutrophils) heteroaggregates was lower in patients with advanced fibrosis/cirrhosis only in stimulated platelets: the median difference was −17.40% (95% CI: −33.30 to −4.90).

### 3.6 Platelet Adhesion Under Flow

**FIGURE 4:**
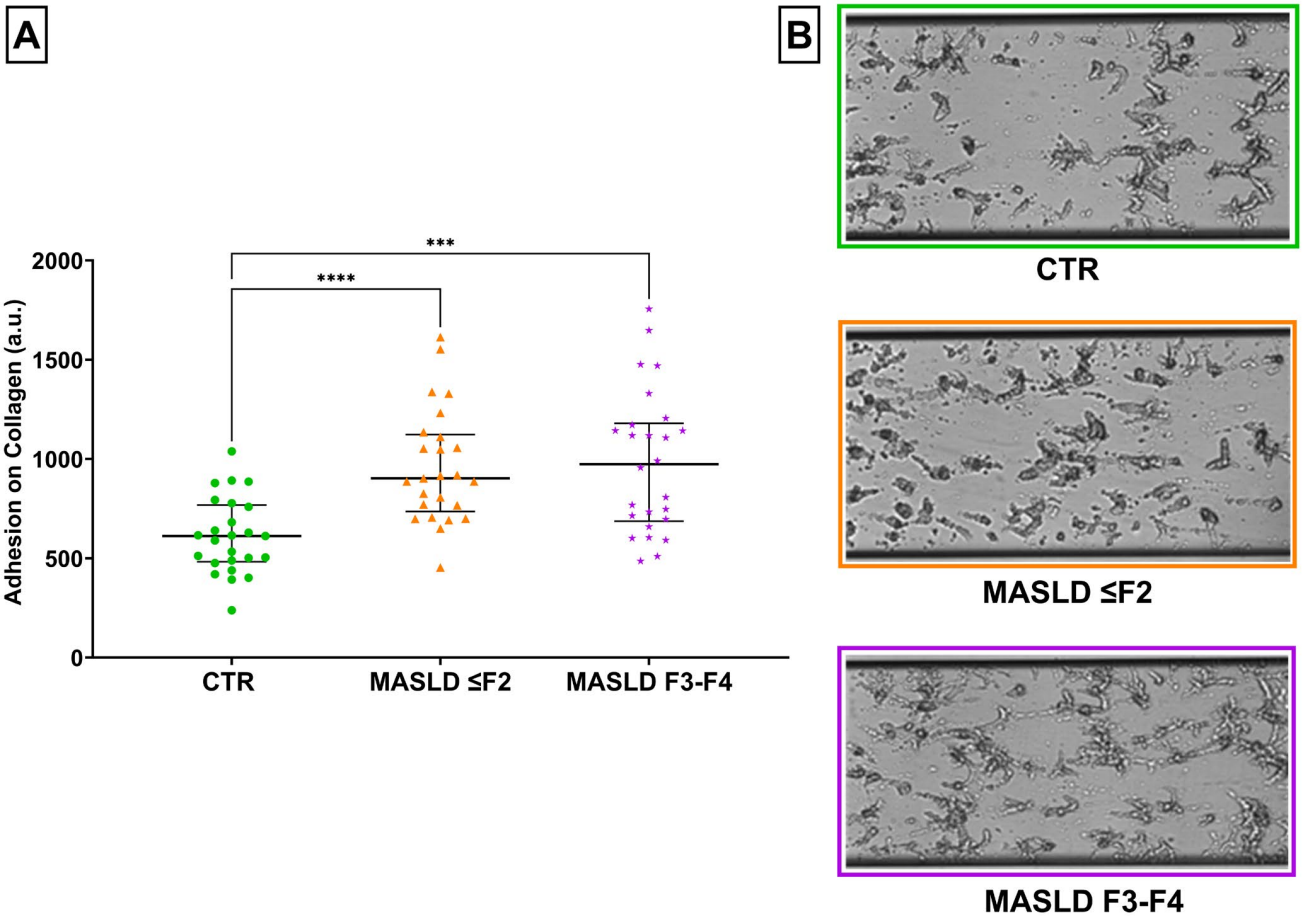
Comparisons of platelets adhesion on a collagen surface in the three studied groups. (A) Platelet adhesion on a collagen surface (a.u.) among the three groups: Control group (CTR), MASLD ≤ F2 group and F3–F4 fibrosis group. (B) Examples of frames acquired using the Microfluidic Cellix platform during the investigation of platelet adhesion to immobilised collagen under basal conditions. From top to bottom: Platelet adhesion in a healthy individual, in a MASLD ≤ F2 patient, and in a patient with advanced fibrosis/liver cirrhosis. ***Kruskal‐Wallis test, *p*‐value < 0.0001; ****Kruskal‐Wallis test, *p*‐value < 0.0001; MASLD F3–F4, patients with advanced fibrosis/cirrhosis group; CTR, control group; MASLD ≤ F2, Metabolic dysfunction‐Associated Steatotic Liver Disease with low liver fibrosis; MASLD F3–F4, Metabolic dysfunction‐Associated Steatotic Liver Disease with advanced liver fibrosis/cirrhosis.

Platelet adhesion under flow was analysed by perfusing with PRP an immobilised collagen surface in a microfluidic device. Subjects belonging to the MASLD ≤ F2 group (median difference: 289.80 a.u., 95% CI: 195.80–458.20) and F3–F4 group (361.10 a.u., 95% CI: 163.40–530.40) displayed higher adhesion under moderate shear stress when compared to the healthy control group (see Figure 4).

### 3.7 Predictors of Platelet Activation in ≤ F2 and F3–F4 Patients

By using liver fibrosis as a readout of the severity of liver disease, we were able to analyse the whole population irrespectively of categorisation based on diagnosis. We considered in separate analyses urinary 11‐dh‐TXB_2_ and plasma sP‐selectin as dependent variables. The multivariate logistic regression analysis showed that urinary 11‐dh‐TXB_2_ higher is associated with both higher LSM values and higher FIB‐4 scores (*β* = 245, *p* < 0.001, and β = 928, *p* < 0.001, respectively) (see also Figure S4).

In addition, the regression analysis found that plasma sP‐selectin was associated with LSM values measured with Fibroscan and FIB‐4 scores (both *p* < 0.001, see also Figure S5).

In two different multivariate logistic regression models, both 11‐dh‐TXB_2_ and sP‐selectin were found independently related to a LSM > 10 kPa (see also Table S1). Similar results were obtained when alternative cut‐offs (e.g., 8 kPa) were applied (see also Supporting Information).

Furthermore, to assess whether portal hypertension might confound these associations, we re‐analysed the data after excluding patients with clinically significant portal hypertension according to the Baveno VII criteria. The results remained consistent, with no significant changes observed in the relationship between platelet activation markers and liver stiffness (see also Supporting Information).

## 4 Discussion

This study aimed to define the platelet profile in MASLD with low fibrosis and advanced fibrosis/cirrhosis. The key finding is that platelet activation correlates with liver fibrosis severity. MASLD ≤ F2 and F3–F4 show distinct platelet patterns: MASLD ≤ F2 is marked by increased platelet adhesiveness and a larger procoagulant platelet subpopulation. In contrast, the F3–F4 group is characterised by low platelet reactivity to stimuli, despite elevated urinary 11‐dh‐TXB_2_ and plasma sP‐selectin, indicating in vivo platelet activation.

In designing the study, we carefully selected patients with metabolic dysfunction as the sole cause of liver disease. We aimed to assess in vivo platelet activation using two validated biochemical markers: urinary 11‐dh‐TXB_2_ and plasma sP‐selectin, which are not sensitive to pre‐analytical artefacts [25]. Although the former quantifies the release of TXA_2_ mostly from activated platelets into the bloodstream and the subsequent urinary elimination of its stable metabolite, the latter measures the shedding of P‐selectin expressed on platelets into the circulation following their activation [26, 27].

Although both biomarkers are primarily of platelet origin, they can also derive from leukocytes and endothelial cells, particularly during inflammation. However, the significant correlation observed between them supports the hypothesis that platelets are the predominant source.

Previous studies have shown increased urinary excretion of stable thromboxane metabolites in patients with liver cirrhosis, which was proportional to the clinical severity of the disease, including liver decompensation [28, 29, 30, 31]. An increase in sP‐selectin has also been shown in patients with liver cirrhosis [32].

We observed that platelet activation increases in patients with liver cirrhosis and is associated with a reduced platelet count. The genesis of thrombocytopenia in cirrhosis is multifactorial, as demonstrated by the normal reticulated platelets: accelerated removal of platelets from the circulation with high turnover and reduced bone marrow production because of reduced hepatic production of thrombopoietin [33]. The results of the present investigation indicate that the increased removal from the circulation is accompanied by platelet activation, possibly upon contact with the target cell or after removal from the circulation. The mechanism responsible for the removal of platelets from the circulation involves platelet and target cell receptors that can activate the platelet. In an experimental murine model of MASLD [34], platelets enter the liver to a greater extent than in controls, and they may contribute to the development of damage and the evolution towards cirrhosis and liver cancer. In this process, the receptor for von Willebrand factor integrin Ib plays a central role. This is not accompanied by a reduction in platelet count nor by platelet activation leading to thromboxane synthesis or secretion, as we have observed. The mechanism of increased platelet removal in liver cirrhosis should therefore be partly different. The main site for platelet removal in liver cirrhosis is the spleen [33] and may have similarities to what was observed and may have similarities to primary and heparin‐induced immune thrombocytopenia in which the excretion of urinary 11‐dh‐TXB_2_ is also increased, as is the plasma concentration of sP‐selectin.

Both indices of in vivo platelet activation correlated linearly with liver fibrosis, based on LSM. The clinical significance of LSM using Fibroscan lies in its role as non‐invasive predictors of disease progression in metabolic liver diseases [20]. Higher fibrosis scores are associated with an increased risk of adverse clinical outcomes, including the progression of cirrhosis and the development of hepatocellular carcinoma [3, 20]. Therefore, the observed correlation between platelet activation markers and liver fibrosis indices provides additional insight into early fibrotic changes and may help identify patients at higher risk for liver‐related complications.

This relationship aligns with experimental evidence showing that activated platelets release growth factors, cytokines and nucleic acids, which contribute to fibrogenesis and the progression to liver cancer [11].

We evaluated different aspects of platelet function to identify both specific and common features in early and advanced liver damage. We assessed platelet adhesion, the surface expression of activated glycoproteins and anionic phospholipids in resting platelets and in response to stimuli, using techniques that allow the investigation of platelets independently of platelet count. Therefore, we did not include platelet aggregation in our study, as it requires a normal platelet count.

Compared to healthy controls, patients with liver disease (either ≤ F2 and F3–F4 groups) showed no differences in P‐selectin expression on the platelet surface under resting conditions, whereas its expression is reduced in response to stimuli. Notably, increased P‐selectin expression in resting platelets is a hallmark of thromboinflammation [35, 36]. Similarly, platelet‐leukocyte aggregates, another characteristic feature of thromboinflammation [35, 36], were not elevated in these patients and are even lower in those with advanced fibrosis/liver cirrhosis.

A distinctive feature of MASLD ≤ F2 patients is the increased presence of platelets binding Annexin V, a marker of platelet procoagulant activity [37]. This binding requires the exposure of anionic phospholipids, which are essential for coagulation factor assembly on the platelet surface. In vitro, strong agonists such as collagen or thrombin can induce this process, usually through immunoreceptor tyrosine‐based activation motif (ITAM) sequences. However, the conditions that lead to the generation of procoagulant platelets in vivo remain unknown.

Recently, a subpopulation of procoagulant platelets, which are also characterised by small volume and decreased expression of the active form of the fibrinogen receptor after stimulation, has been described in patients with diabetes mellitus [37, 38]. Specific platelet subpopulations can be identified based on phenotype under resting conditions and their functional response to stimulus. In the MASLD ≤ F2 patients, the presence of Annexin V binding, which is increased in vitro by platelet stimulation with a strong agonist, is associated with reduced expression of the active fibrinogen receptor compared to control, suggesting that procoagulant platelets activity may characterise this clinical condition [38].

In both patient populations, particularly those with advanced fibrosis/liver cirrhosis, a distinct pattern of reduced responsiveness to platelet agonists has emerged. Similar impairments in platelet activation have been reported in previous studies using platelet aggregation or flow cytometry, and were linked to increased urinary excretion of thromboxane metabolites. This suggests that the reduced response to stimuli reflects a specific inhibitory mechanism at the signalling level [39, 40].

A reduced expression of the active form of the fibrinogen receptor in response to stimuli has been observed in various clinical and experimental conditions, including viral and bacterial infections or aseptic inflammation. This phenomenon is thought to result from either receptor masking or the activation of specific inhibitory signalling pathways [41].

The mechanisms behind thrombocytopenia in liver cirrhosis are yet to be defined by future studies. However, the observed reduced expression of the active fibrinogen receptor may not lead to reduced platelet aggregation in vivo [42, 43].

Although in MASLD ≤ F2 and especially in F3–F4 patients there is evidence of reduced response to agonists, platelet adhesion to immobilised collagen under flow conditions is increased. Collagen is the ligand that causes stable platelet adhesion to the receptor constitutively expressed on the surface of platelets (complex Ia/IIa‐IX). This is the mechanism of stable platelet adhesion in the presence of endothelial damage, further activating platelets. The increase in platelet adhesion may involve mechanisms that lead to enhanced platelet activation in vivo, possibly resulting in translocation to the liver in MASLD ≤ F2 and platelet removal from circulation in advanced fibrosis/liver cirrhosis [33].

Considering the platelet profile from the present study and the available evidence, an imbalance of haemostasis towards a prothrombotic condition is emerging in chronic liver disease [7, 44, 45].

Coagulation factors, whose alteration particularly affects the haemostatic balance in liver cirrhosis, and markers of inflammation were not analysed in the present study, limiting the mechanistic insights.

In diabetes mellitus, treatment with low‐dose aspirin substantially reduced urinary excretion of 11‐dh‐TXB_2_, confirming its prevalent platelet origin and demonstrating its role in the pathogenesis of cardiovascular events [46, 47].

Concerning the liver disease, a similar demonstration was obtained in experimental and clinical studies with antiplatelet drugs [11, 48], which have shown that aspirin and thienopyridines decrease the progression of MASLD towards cirrhosis and liver cancer. This confirms the hypothesis of a pathogenetic role of platelets while identifying a target for the prevention of disease progression and suggesting a possible protective role towards thrombotic events related to the increased cardiovascular risk [49].

We acknowledge the absence of a separate analysis between patients with stage F3 fibrosis and those with F4 cirrhosis as a limitation of our study. Due to the relatively small number of subjects in each subgroup, it was not feasible to perform statistically meaningful comparisons. Future studies with larger cohorts are warranted to allow for a more granular stratification of disease severity, which may uncover further insights into the dynamic changes in platelet function across progressive stages of MASLD.

## 5 Conclusions

In conclusion, our study of platelet phenotype and function in MASLD ≤ F2 and F3–F4 fibrosis reveals both common features and significant differences. Platelet activation in both conditions correlates with fibrosis severity, and in advanced fibrosis/cirrhosis, with thrombocytopenia severity. In MASLD ≤ F2, increased platelet adhesiveness and procoagulant platelets may contribute to a prothrombotic state, as seen in other high cardiovascular risk conditions. Particularly in F3–F4 patients, activated platelets likely contribute to disease progression. These findings support the hypothesis that platelets play a role in chronic liver disease progression and suggest that antiplatelet therapies could help prevent the progression of liver fibrosis and tumourigenesis. Further clinical research is needed to test this hypothesis.

## Author Contributions

M.C. and M.Z. equally contributed to the study concept and design, data collection, laboratory experiments, statistical analysis and drafting of the initial manuscript. A Meneguzzi contributed to the laboratory experiments. A Mantovani contributed to the data collection. D.S. and P.M. supervised the project, reviewed the study concept and design and contributed to the review and editing of the manuscript. A.D. assisted with the statistical analyses and participated in drafting the manuscript. All authors had full access to the study data and had final responsibility for the decision to submit the manuscript for publication.

## Conflicts of Interest

P.M. received public funds for the independent research of the Azienda Ospedaliera Universitaria Integrata (AOUI) Verona. All the other authors declare no conflicts of interest.

## Supporting information


Data S1.

## Data Availability

The data that support the findings of this study are available on request from the corresponding author. The data are not publicly available due to privacy or ethical restrictions.
