# Supplementary material for: Platelet Functional Profile Is Altered in Metabolic Dysfunction‐Associated Steatotic Liver Disease
Source: Liver Int. 2025 Jul 28;45(8):e70231. doi: 10.1111/liv.70231 (PMC12302667; doi:10.1111/liv.70231)
Supplement: Supplementary file 1 — Data S1. [file LIV-45-0-s001.docx]

**Supplementary data.**

**Supplementary Figure 1. Distribution of platelet index across control, MASLD ≤F2, and MASLD F3-F4 groups.**


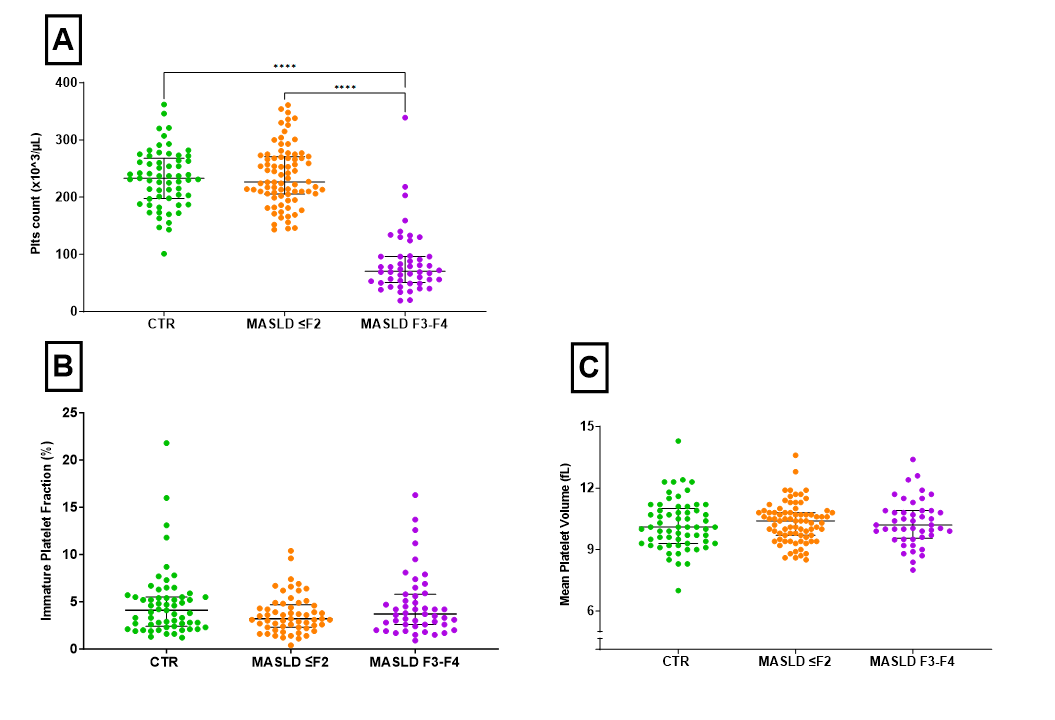


Comparison of platelet indexes across three groups: control, MASLD ≤F2, and MASLD F3-F4. A) Platelet count (x10^3^/µL) among the 3 groups: control group (CTR), MASLD with fibrosis ≤F2, and MASLD with fibrosis F3-F4. B) Immature platelet fraction (%) among the 3 groups: CTR, MASLD ≤F2, and MASLD F3-F4. C) Mean platelet volume (fL) among the three groups: CTR, MASLD ≤F2, and MASLD F3-F4.

*Legend:* *****, Kruskal-Wallis test, p-value <0.0001; CTR, healthy subjects in the control group; MASLD ≤F2, Metabolic dysfunction-Associated Steatotic Liver Disease with low liver fibrosis; MASLD F3-F4, Metabolic dysfunction-Associated Steatotic Liver Disease with advanced liver fibrosis/cirrhosis Plts, platelets.*

**Supplementary Figure 2. Distribution of P-selectin expression in resting and activated platelets, among the control, MASLD ≤F2, and F3-F4 groups.**


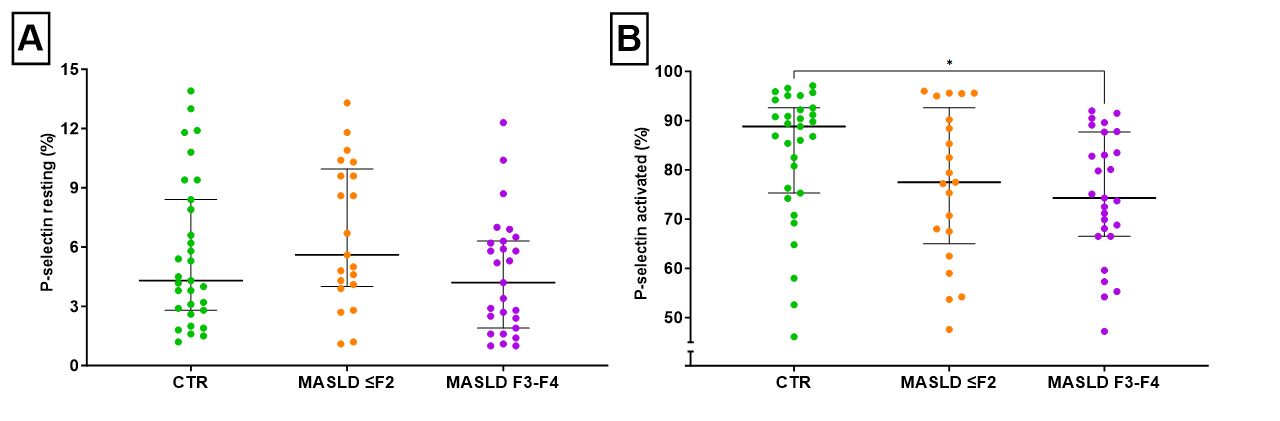


A) P-selectin expression (%) considering platelets in resting condition among the three groups: control (CTR), MASLD ≤F2, and MASLD F3-F4 group.

B) P-selectin expression (%) considering platelets in activated condition, among the three group: CTR, MASLD ≤F2, and MASLD F3-F4 group. TRAP-6 20µM was used as agonist.

*Legend: *, Kruskal-Wallis test, p-value <0.05; CTR, control group; MASLD ≤F2, Metabolic dysfunction-Associated Steatotic Liver Disease with low liver fibrosis; MASLD F3-F4, Metabolic dysfunction-Associated Steatotic Liver Disease with advanced liver fibrosis/cirrhosis.*

**Supplementary Figure 3. Distribution of hetero-aggregates expressions in resting and activated platelets, among the control, MASLD ≤F2, and MASLD F3-F4 group.**


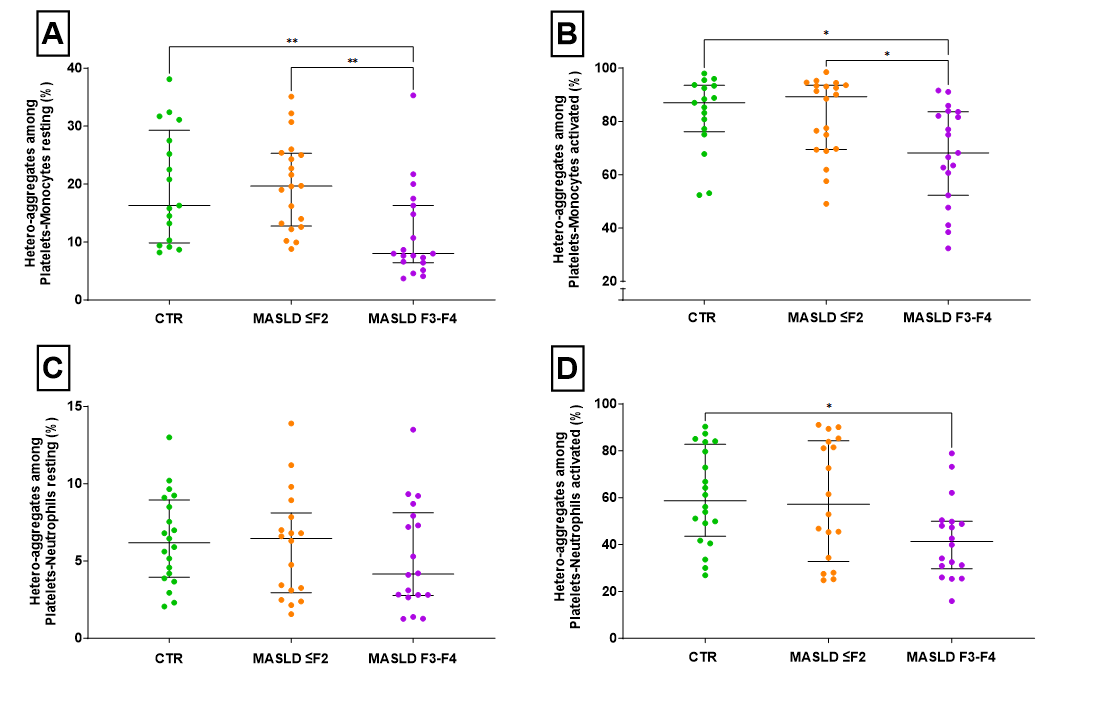


A) Distribution of platelets-monocytes hetero-aggregates (%), considering resting platelets, across the three groups: control group (CTR), MASLD ≤F2, and MASLD F3-F4 group. B) Distribution of platelets-monocytes hetero-aggregates (%), considering activated platelets, across the three groups: CTR, MASLD ≤F2, and MASLD F3-F4 group. TRAP-6 20µM was used as agonist.

C) Distribution of platelets-neutrophils hetero-aggregates (%), considering resting platelets, across the three groups: CTR, MASLD ≤F2, and MASLD F3-F4 group.

D) Distribution of platelets-neutrophils hetero-aggregates (%), considering activated platelets, across the three groups: CTR, MASLD ≤F2, and MASLD F3-F4 group. TRAP-6 20µM was used as agonist.

*Legend: *, Kruskal-Wallis test, p-value <0.05; **, Kruskal-Wallis test, p-value <0.01; CTR, control group; MASLD ≤F2, Metabolic dysfunction-Associated Steatotic Liver Disease with low liver fibrosis; MASLD F3-F4, Metabolic dysfunction-Associated Steatotic Liver Disease with advanced liver fibrosis/cirrhosis.*

**Supplementary Figure 4.**


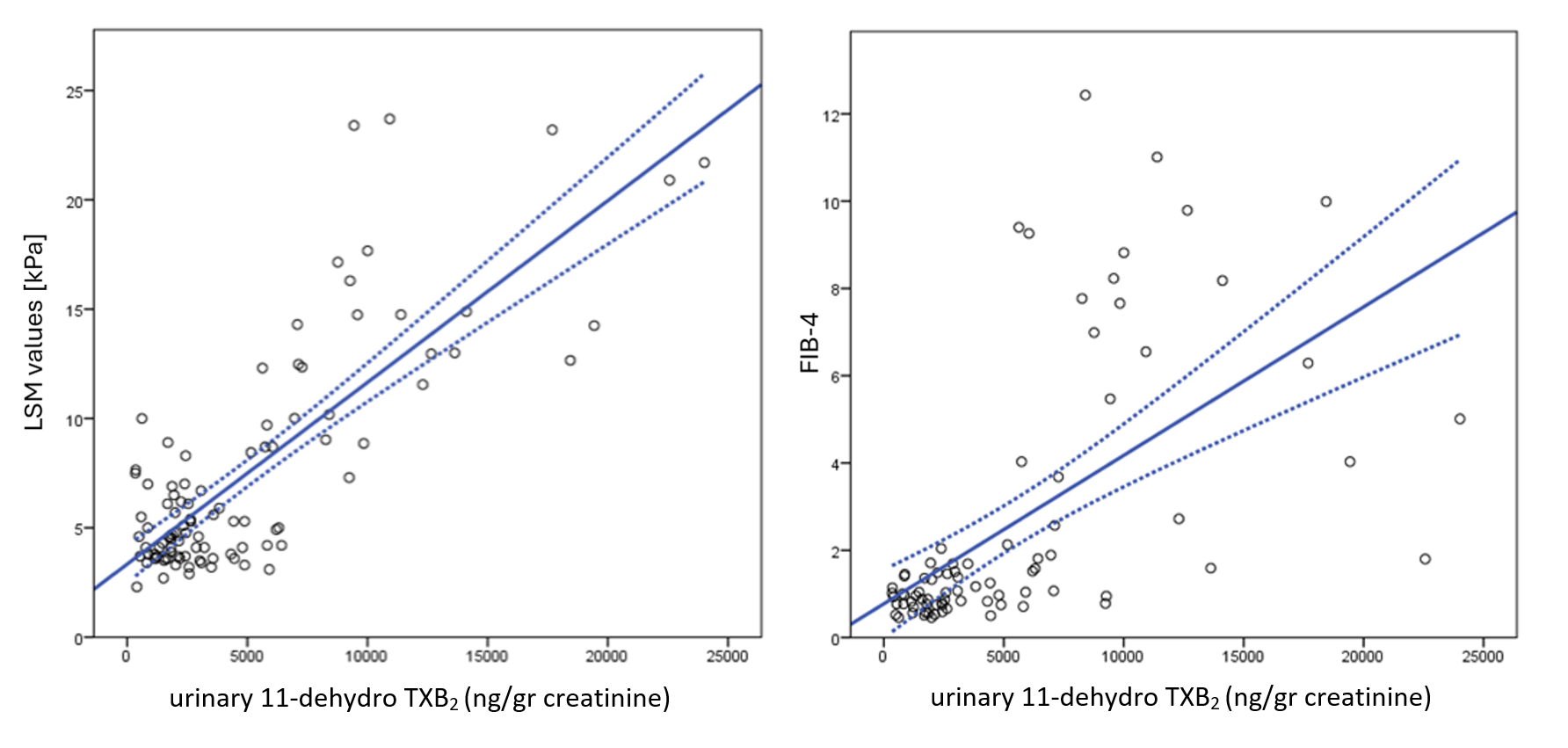


Solid lines represent the line of best fit; dotted lines represent the 95% confidence interval.

*Legend: FIB-4, Fibrosis-4 index; LSM, liver stiffness measurement.*

**Supplementary Figure 5.**


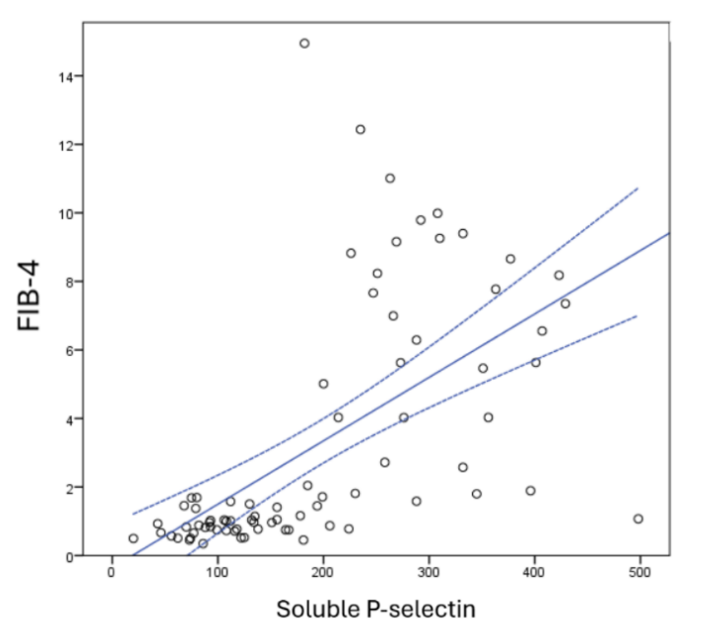

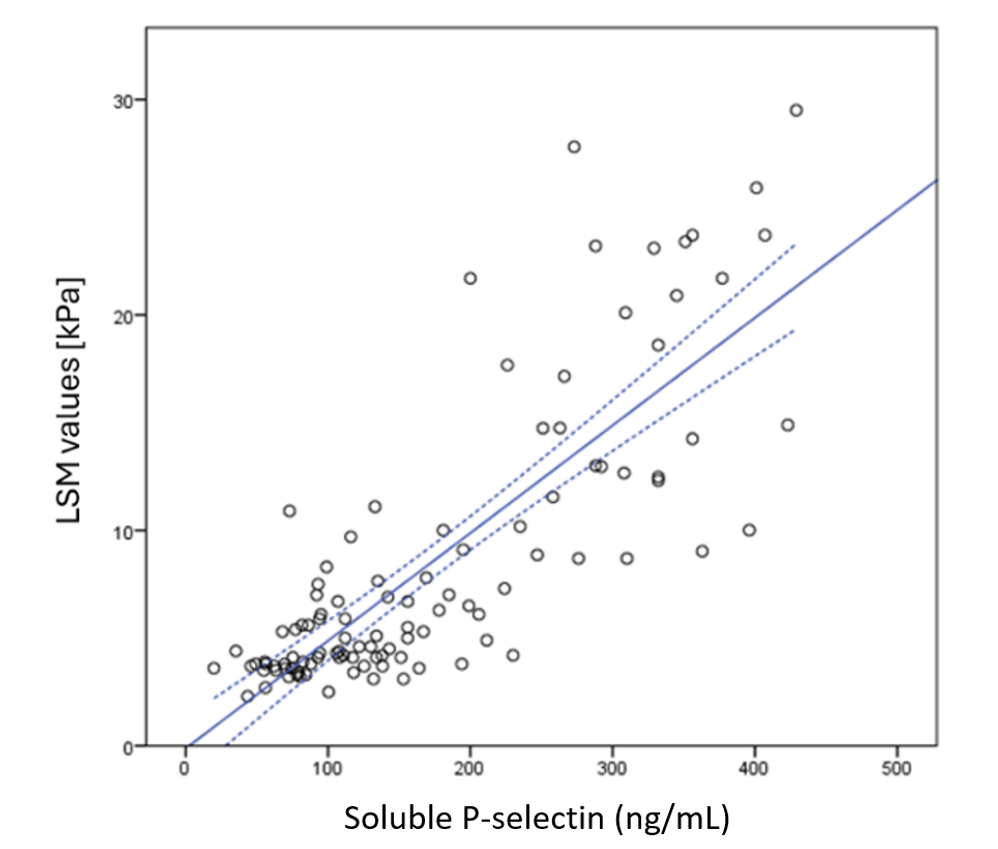


Solid line represents the line of best fit; dotted lines represent the 95% confidence interval. *Legend: LSM, liver stiffness measurement.*

**Supplementary Table 1. Univariate and multivariate logistic regression analyses of potential predictors for Fibroscan >10 kPa.**

| **Variable** | **Univariate analysis** | |
| --- | --- | --- |
|  | **OR (CI 95%)** | **p-value** |
| Age | 1.08 (1.04 – 1.12) | **<0.001** |
| Sex, female | 0.48 (0.22 – 1.06) | 0.07 |
| Obesity | 1.64 (0.65 – 4.17) | 0.30 |
| Hypertension | 2.22 (1.05 – 4.69) | **0.04** |
| Dyslipidaemia | 1.21 (0.57 – 2.56) | 0.62 |
| Type 2 diabetes mellitus | 12.84 (5.05 – 32.62) | **<0.001** |
| Haemoglobin | 0.96 (0.94 – 0.98) | **<0.001** |
| MCV | 1.12 (1.06 – 1.19) | **<0.001** |
| WBC | 0.89 (0.75 – 1.06) | 0.21 |
| PLTs | 0.98 (0.97 – 0.99) | **<0.001** |
| PDW | 0.96 (0.81 – 1.13) | 0.59 |
| MPV | 1.12 (0.81 – 1.57) | 0.49 |
| IPF | 1.07 (0.96 – 1.19) | 0.23 |
| Albumin | 0.84 (0.75 – 0.93) | **<0.001** |
| Total bilirubin | 1.13 (0.99 – 1.29) | 0.06 |
| Ferritin | 1.002 (1.001 – 1.01) | **0.03** |
| AST | 1.03 (1.01 – 1.05) | **<0.001** |
| ALT | 1.01 (0.99 – 1.02) | 0.44 |
| Total cholesterol | 0.98 (0.97 – 0.99) | **<0.001** |
| LDL cholesterol | 0.97 (0.96 – 0.99) | **<0.01** |
| HDL cholesterol | 0.99 (0.95 – 1.02) | 0.43 |
| Triglycerides | 0.99 (0.99 – 1.01) | 0.59 |
| Glucose | 1.03 (1.01 – 1.04) | **<0.001** |
| 11-dh-TxB2 | 1.001 (1.001 – 1.001) | **<0.001** |
| sP-selectin | 1.02 (1.02 – 1.03) | **<0.001** |
| **Variable** | **Multivariate analysis – Model 1** | |
|  | **OR (CI 95%)** | **p-value** |
| Sex, female | 0.19 (0.01 – 2.65) | 0.22 |
| Age | 1.06 (0.96 – 1.16) | 0.26 |
| Type 2 diabetes mellitus | 17.64 (1.51 – 206.34) | **0.02** |
| PLTs | 1.02 (0.99 – 1.04) | 0.12 |
| 11-dhTx-B2 | 1.001 (1.0003 – 1.001) | **<0.01** |
| **Variable** | **Multivariate analysis – Model 2** | |
|  | **OR (CI 95%)** | **p-value** |
| Sex, female | 0.55 (0.09 – 3.32) | 0.51 |
| Age | 0.94 (0.86 – 1.02) | 0.12 |
| Type 2 diabetes mellitus | 1.51 (0.19 – 11.78) | 0.69 |
| PLTs | 0.99 (0.99 – 1.01) | 0.59 |
| sP-selectin | 1.03 (1.01 – 1.05) | **<0.01** |

Numbers in bold represent statistical significance.

Two distinct multivariate logistic regression models are considered.

*Legend: 11-dh-TxB2, 11-dheydro-Tromboxane B_2_; ALT, alanine aminotransferase; AST, aspartate aminotransferase; DM, type 2 diabetes mellitus; IPF, immature platelet fraction; MCV, mean corpuscolate volume; MPV, mean platelet volume; PDW, platelet distribution width; PLTs, platelet count; sP-selectin, soluble P-selectin; WBC, white blood cells.*

**Supplementary Figure 6.** **Distribution of P-selectin (ng/mL) and urinary 11-dehydro-thromboxane B2 (ng/g creatinine) across study groups, including MASLD F3-F4 patients stratified by Fibroscan stiffness and platelet count.**


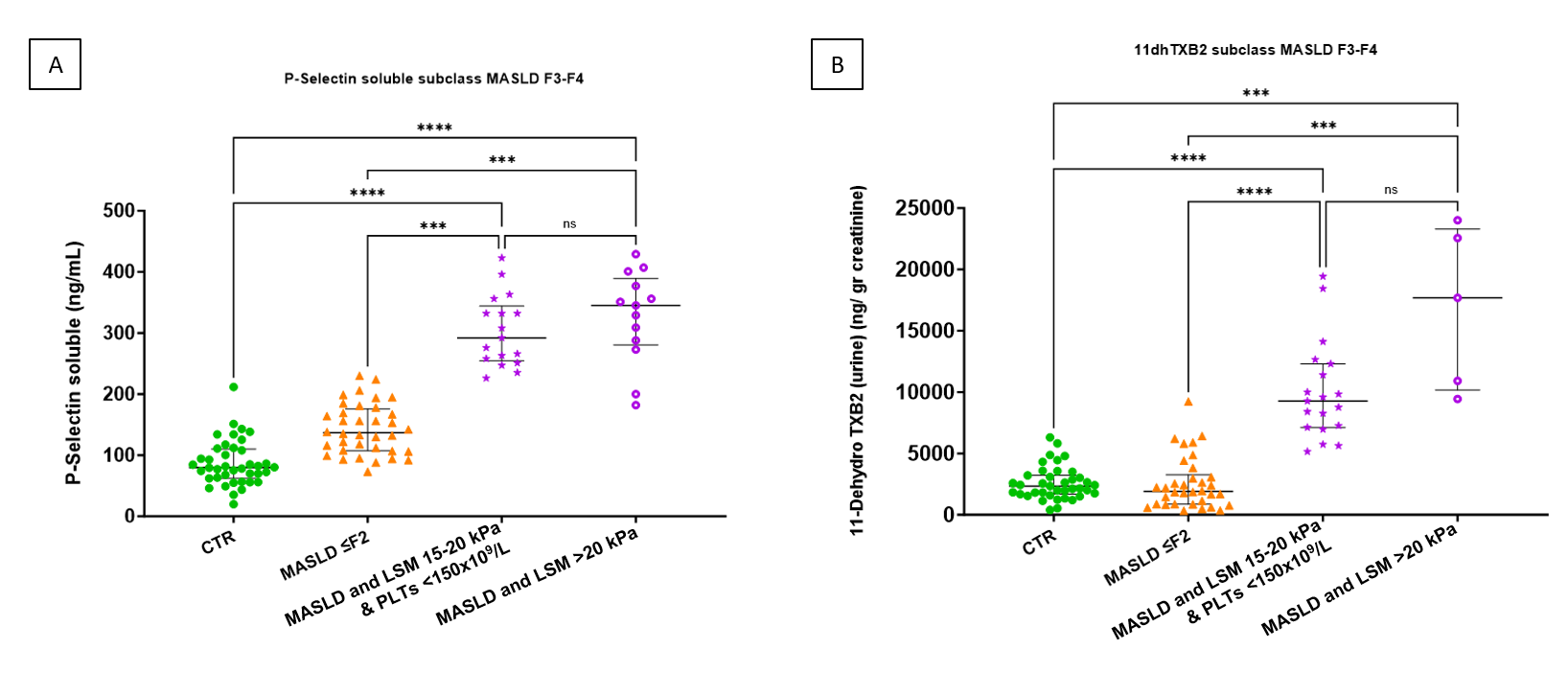


Panel A shows P-selectin levels in healthy controls, MASLD ≤F2, MASLD with liver stiffness measurement (LSM) between 15–20 kPa and platelet count <150×10⁹/L, and MASLD with LSM >20 kPa. Panel B shows urinary 11-dehydro-TXB2 levels in the same subgroups.

*Legend: ***, p-value <0.001; ****, p-value <0.0001; ^ns^, not significant; CTR, control group; LSM, liver stiffness measurement; MASLD, Metabolic dysfunction-Associated Steatotic Liver Disease; MASLD ≤F2, Metabolic dysfunction-Associated Steatotic Liver Disease with low liver fibrosis; PLTs, platelet count.*

**Supplementary Figure 7.** **Platelet-to-Leukocyte Ratio in healthy controls and MASLD patients, according to disease severity and Fibroscan stratification.**


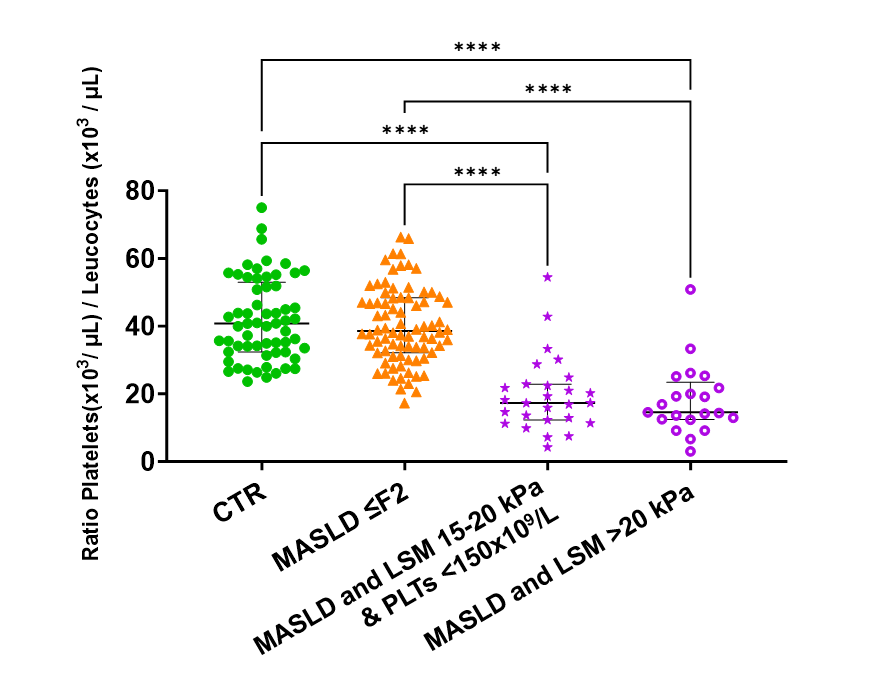


Ratio between platelet count (×10³/µL) and leukocyte count (×10³/µL) across study groups, including MASLD F3–F4 subgroups stratified by liver stiffness and thrombocytopenia.

The analysis compares healthy controls (CTR), MASLD ≤F2, MASLD with liver stiffness measurement (LSM) between 15–20 kPa and platelet count <150×10⁹/L, and MASLD with LSM >20 kPa. A significant decrease in the platelet-to-leukocyte ratio is observed in patients with advanced disease stages.

*Legend: ****, p-value <0.0001; CTR, control group; LSM, liver stiffness measurement; MASLD, Metabolic dysfunction-Associated Steatotic Liver Disease; MASLD ≤F2, Metabolic dysfunction-Associated Steatotic Liver Disease with low liver fibrosis; PLTs, platelet count.*

**Supplementary Figure 8.** **Platelet activation markers and liver stiffness in healthy controls and MASLD patients, stratified by fibrosis stage and diabetes status.**


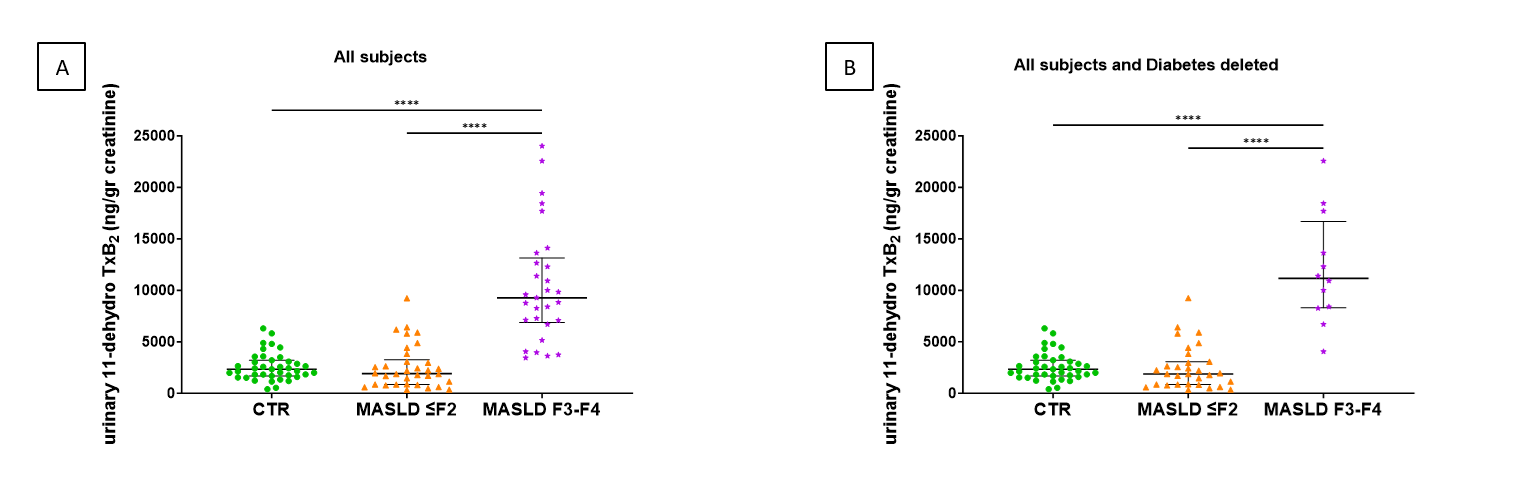


**Total population**

**Only subjects without DM2**

A

B


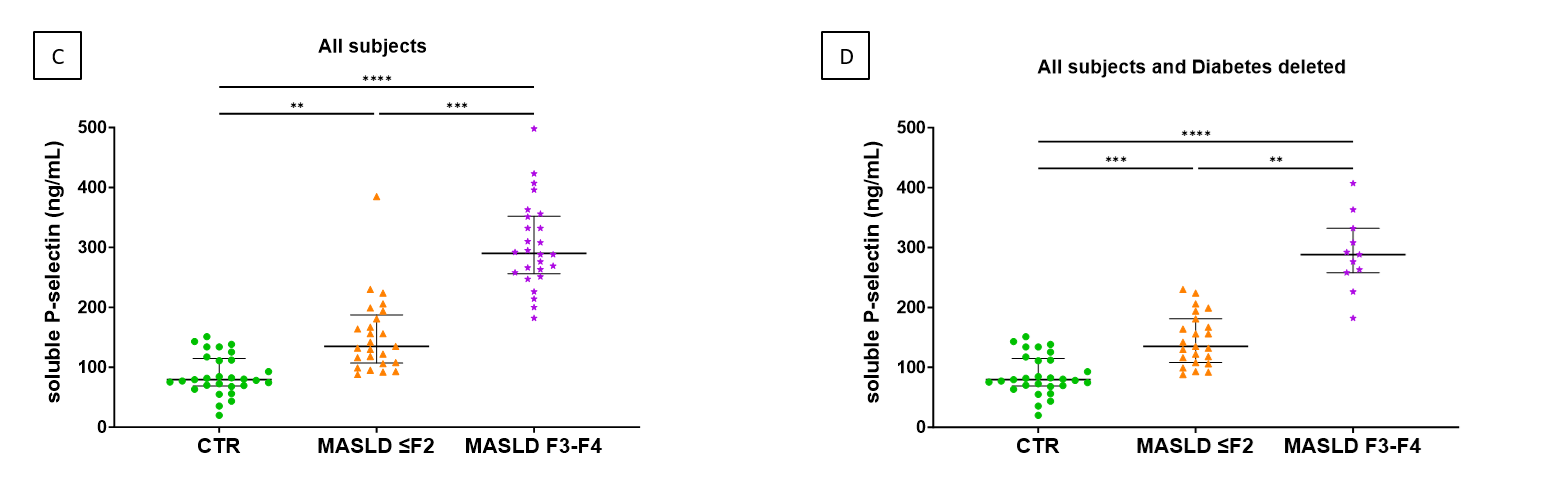


**Total population**

**Only subjects without DM2**

C

D


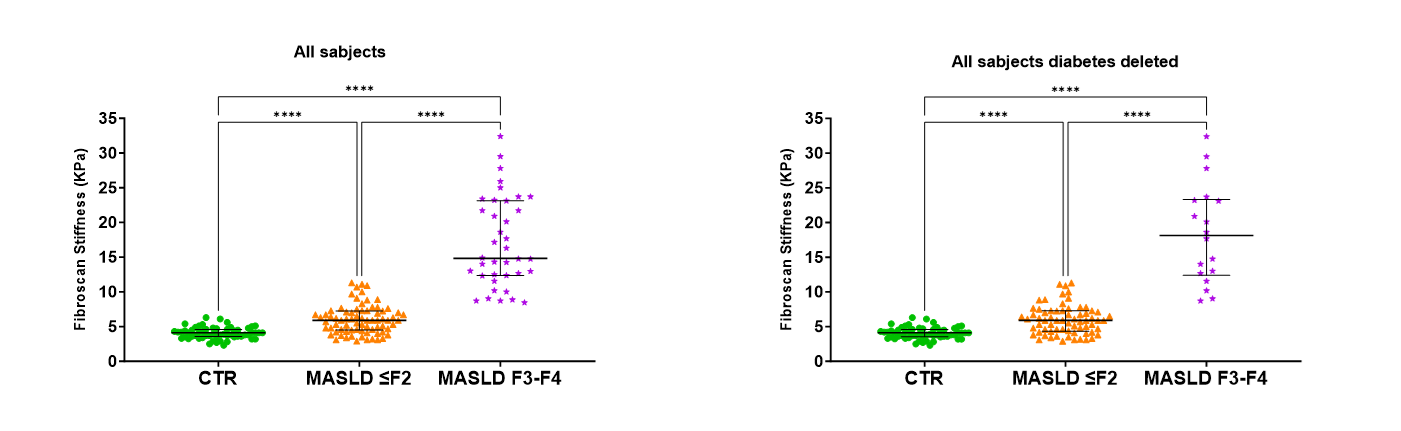


**Total population**

**Only subjects without DM2**

E

F


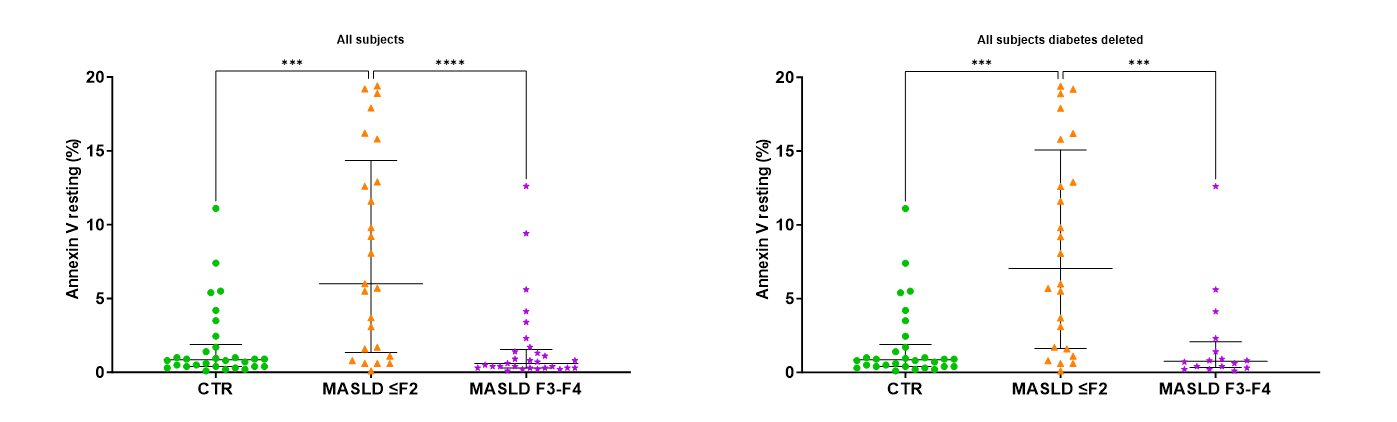


**Total population**

**Only subjects without DM2**

G

H


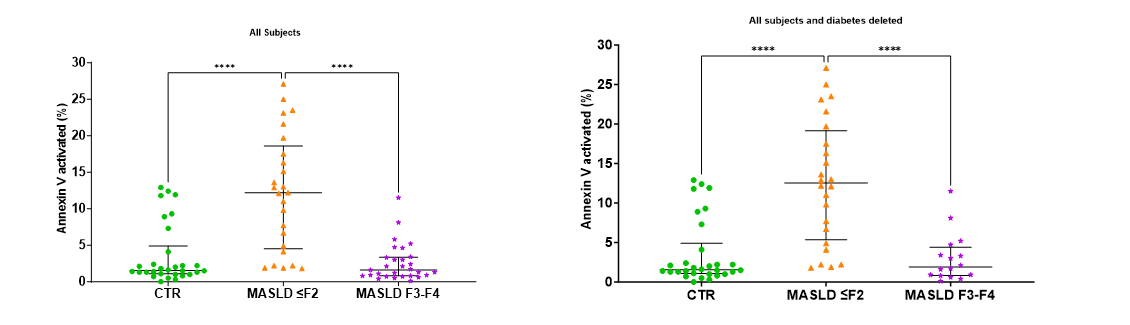


**Total population**

**Only subjects without DM2**

I

J

Comparative analysis of platelet activation and liver stiffness parameters in the overall study population (Panels A, C, E, G, I) and in the subgroup without type 2 diabetes mellitus (Panels B, D, F, H, J), stratified into three groups: healthy controls, MASLD with ≤F2, and MASLD with advanced fibrosis (F3–F4). Panels A-B show the analyses for urinary 11-dehydro-thromboxane B_2_ (ng/g creatinine); panels C-D show the analyses for soluble P-selectin (ng/mL); panels E-F show the analyses for liver stiffness measurement using Fibroscan (kPa); panels G-H show the analyses for Annexin V activity in resting state (%); panels I-J show the analyses for Annexin V activity in activated state, after stimulation (%).

*Legend: **, p-value < 0.01; ***, p-value <0.001; ****, p-value < 0.0001; CTR, control group; DM2, type 2 diabetes mellitus; MASLD ≤F2, Metabolic dysfunction-Associated Steatotic Liver Disease with low liver fibrosis; MASLD F3-F4, Metabolic dysfunction-Associated Steatotic Liver Disease with advanced liver fibrosis/cirrhosis.*

**Supplementary Figure 9. Correlations between 11-dehydro-tromboxane B_2_ and P-selectin with spleen size**.


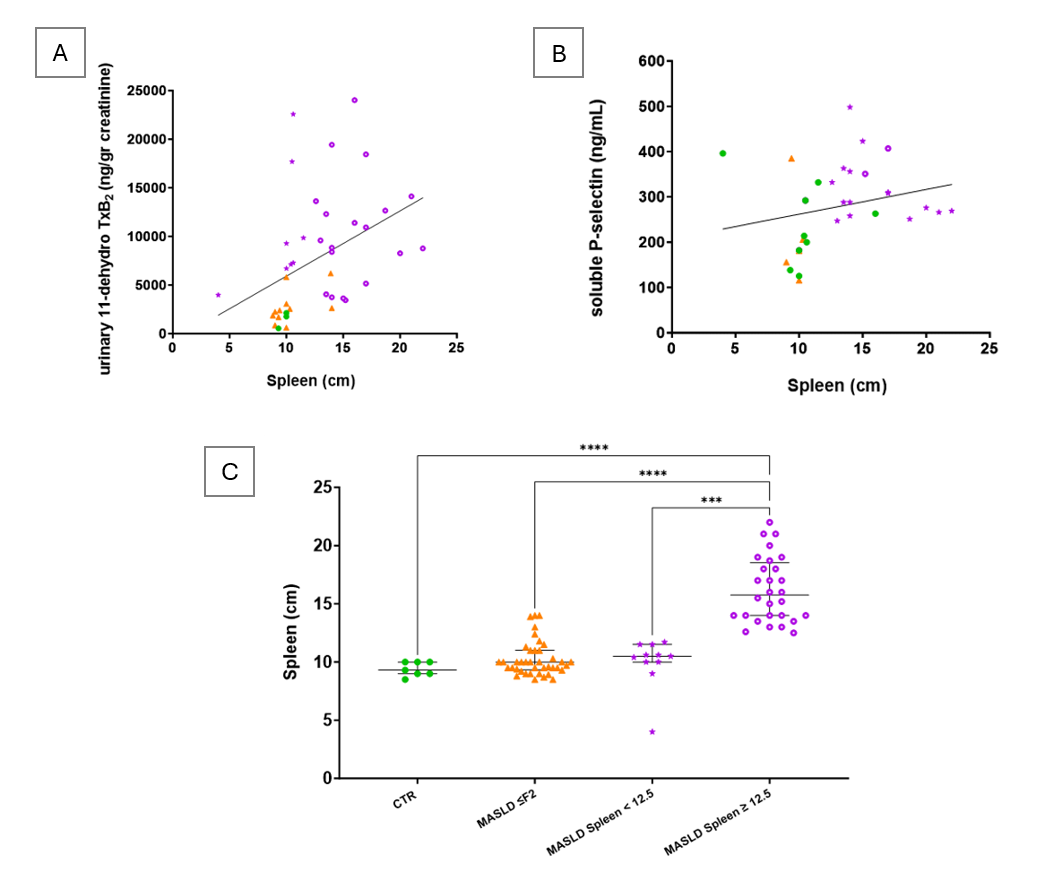


Panel A shows the correlation between 11-dehydro-tromboxane B_2_ and spleen size in the study population. Panel B displays the correlation between soluble P-selectin and spleen among the study population. Panel C presents a comparative analysis of spleen size across study groups, including MASLD F3-F4 patients further stratified by a spleen size cut-off of 12.5 cm.

*Legend: CTR, control group; MASLD ≤F2, Metabolic dysfunction-Associated Steatotic Liver Disease with low liver fibrosis; MASLD Spleen < 12.5, Metabolic dysfunction-Associated Steatotic Liver Disease with advanced liver fibrosis/cirrhosis and a spleen size less than 12.5 cm; MASLD Spleen ≥ 12.5, Metabolic dysfunction-Associated Steatotic Liver Disease with advanced liver fibrosis/cirrhosis and a spleen size more or equal to 12.5 cm.*
